# Large extracellular vesicles and blebbisomes in cancer: emerging and translational opportunities highlights

**DOI:** 10.1186/s12964-026-02670-0

**Published:** 2026-01-21

**Authors:** Xin Zhang, Jianan Zhe, Changling Duan, Xinyi Wei, Zhong He, Chengran Shi, Lei Yuan, Hao Wen, Wei Bao, Qiong Fan

**Affiliations:** 1 Department of Gynecologic Oncology, Shanghai Key Laboratory of Embryo Original Diseases, The International Peace Maternity and Child Health Hospital, Shanghai Jiao Tong University School of Medicine, No. 910, Hengshan Road, Shanghai, 200030 China; 2 Department of Gynecologic Oncology, Shanghai Geriatric Medical Center, Shanghai, 201100 China; 3 Department of Obstetrics and Gynecology, SongJiang Matertity and Child Health Hospital, No. 1010, Xilin North Road, Shanghai, 201620 China; 4 Department of Gynecologic Oncology, Obstetrics and Gynecology Hospital, Fudan University, Shanghai, 200001 China; 5 Department of Gynecologic Oncology, Fudan University Shanghai Cancer Center, Shanghai, 200032 China; 6 Department of Gynecology, Shanghai Key Laboratory of Maternal Fetal Medicine, Shanghai Institute of Maternal-Fetal Medicine and Gynecologic Oncology, Shanghai First Maternity and Infant Hospital, School of Medicine, Tongji University, Shanghai, 200092 China; 7 Department of Obstetrics and Gynecology, Shanghai General Hospital, Shanghai Jiao Tong University School of Medicine, No. 100, Haining Road, Shanghai, 200080 China

**Keywords:** Large extracellular vesicles, Blebbisomes, Microvesicles, Apoptotic bodies, Large oncosomes, Migrasomes, Exophers, Midbody remnants, Biogenesis and regulation, Cargo and function, Local invasion, Tumor microenvironment, Immune evasion, Tumor immunology, Liquid biopsy, Drug delivery vehicles, Immunotherapy targets

## Abstract

Large extracellular vesicles (lEVs), particularly the recently identified blebbisomes, are emerging as critical mediators of tumor progression and intercellular communication. Compared with small vesicles, lEVs exhibit pronounced heterogeneity in size, cargo composition, and mechanisms of biogenesis. While EVs of all sizes can carry proteins, nucleic acids, lipids, and metabolites, lEVs more frequently encapsulate bulky cargos—including intact organelles such as mitochondria—reflecting their size-enabled loading capacity rather than a feature unique to lEVs. These characteristics position lEVs as key regulators of immune responses, metabolic reprogramming, and the establishment of pre-metastatic niches within the tumor microenvironment. Blebbisomes, distinguished by their dynamic membrane behavior, bidirectional cargo transfer, and high expression of immunosuppressive molecules, represent a novel paradigm in extracellular communication. However, challenges persist in defining lEV subtypes, achieving efficient purification and isolation, and accurately tracking their behavior in vivo. This review systematically summarizes recent advances in lEV research in tumor biology, highlights the distinctive functions of blebbisomes, and examines their translational potential in diagnostics and therapy. Key knowledge gaps are identified, including the need for single-vesicle multi-omics, advanced lipidomics, and engineered analytical platforms. We advocate for expanded investigation into lEVs as promising targets and tools in precision oncology.

## Highlights

- Highlights the roles of lEVs and blebbisomes in tumor immunity and intercellular communication.
- Defines blebbisomes as dynamic, immunosuppressive“pseudo-cellular” vesicles.
- Summarizes recent advances in lEV biology and tumor microenvironment regulation.
- Identifies key challenges in lEV classification, purification, and in vivo tracking.
- Proposes multi-omics and bioengineering strategies for lEV-based cancer diagnostics and therapy.

## Introduction

Tumor initiation, progression, and metastasis are complex, multifactorial processes driven by dynamic interactions between cancer cells and their surrounding microenvironment. In recent years, extracellular vesicles (EVs) have emerged as essential mediators of intercellular communication and have become a major focus of cancer research [1–4]. According to the MISEV 2023 guidelines, EVs are categorized into small extracellular vesicles (sEVs, < 200 nm) and large extracellular vesicles (lEVs, > 200 nm), with 200 nm serving as the cutoff. Although this classification is not universally accepted across the academic community, the guidelines underscore the importance of standardizing EV size definitions; therefore, this study adopts the recommended classification system [5, 6]. While the mechanisms of sEVs have been relatively well characterized, the functional roles of lEVs in tumor progression have long been underestimated. Emerging evidence indicates that lEVs are not simply cellular “excreta,” but instead are structurally complex, functionally diverse, and biologically active entities. Although lEVs display enhanced cargo-carrying capacity, enriched surface molecule expression, and a greater ability to encapsulate organelles, these features are shared with sEVs, which have been more extensively studied. Characteristics once attributed primarily to sEVs are now recognized in lEVs as well, highlighting their functional overlap [7, 8]. Moreover, EVs of various sizes participate in material transfer, immune modulation, and pre-metastatic niche formation. lEVs may contribute to these processes through mechanically distinct, size-enabled mechanisms—such as higher-force cellular interactions and organelle shuttling—depending on their subtype and biological context [7].

In 2025, Jeppesen et al. first described and named a novel subtype of lEVs—blebbisomes—characterized by their large diameter (10–20 μm), the presence of multiple organelles, absence of a nucleus, and continuous “blebbing” motility coupled with multifaceted, cell-like behaviors. These features suggest that blebbisomes may act as “signal integration hubs” within the tumor microenvironment, playing critical roles in the bidirectional uptake and release of biological information [9]. Studies have shown that, beyond differences in size, lEVs and blebbisomes differ substantially in cargo composition. These cargoes include, but are not limited to, nucleic acids, proteins, lipids, and various small molecules, collectively contributing to intercellular communication, pathological signal transduction, and cellular responses to environmental stimuli [10]. Notably, because of their large size and unique structural features, blebbisomes can transport a greater load of functional molecules than traditional EVs, making them indispensable contributors to tumor development [9].

Additionally, although sEVs have dominated the EV field for decades due to their relative ease of isolation and characterization, lEVs are increasingly recognized as potentially superior vehicles for both diagnostic and therapeutic applications. Unlike sEVs, lEVs possess several unique advantages, including: (i) the capacity to package entire functional organelles such as mitochondria [11], (ii) the ability to reduce cancer cell migration [12], and (iii) the potential to transport complex cargo assemblies, such as organelle fragments and multiple vesicles simultaneously. These features position lEVs as promising candidates in applications where sEVs may be limited, particularly in settings that require the transfer of large functional units or complex therapeutic payloads.

The discovery of blebbisomes not only challenges traditional EV classifications but also provides new insights into their roles in tumor heterogeneity, immune regulation, and metabolic adaptation. This review systematically summarizes current advances in research on lEVs and blebbisomes within the context of tumor biology. Topics include their classification, mechanisms of biogenesis and release, cargo composition, and functional roles during various stages of tumor progression, as well as their isolation methods and translational potential in clinical applications. Moreover, we address key challenges in the field and outline future research directions, with the goal of providing a theoretical foundation and reference framework for a deeper understanding of lEVs and blebbisomes and their potential roles in tumor diagnosis and therapy.

## Classification of lEVs

**Fig. 1:**
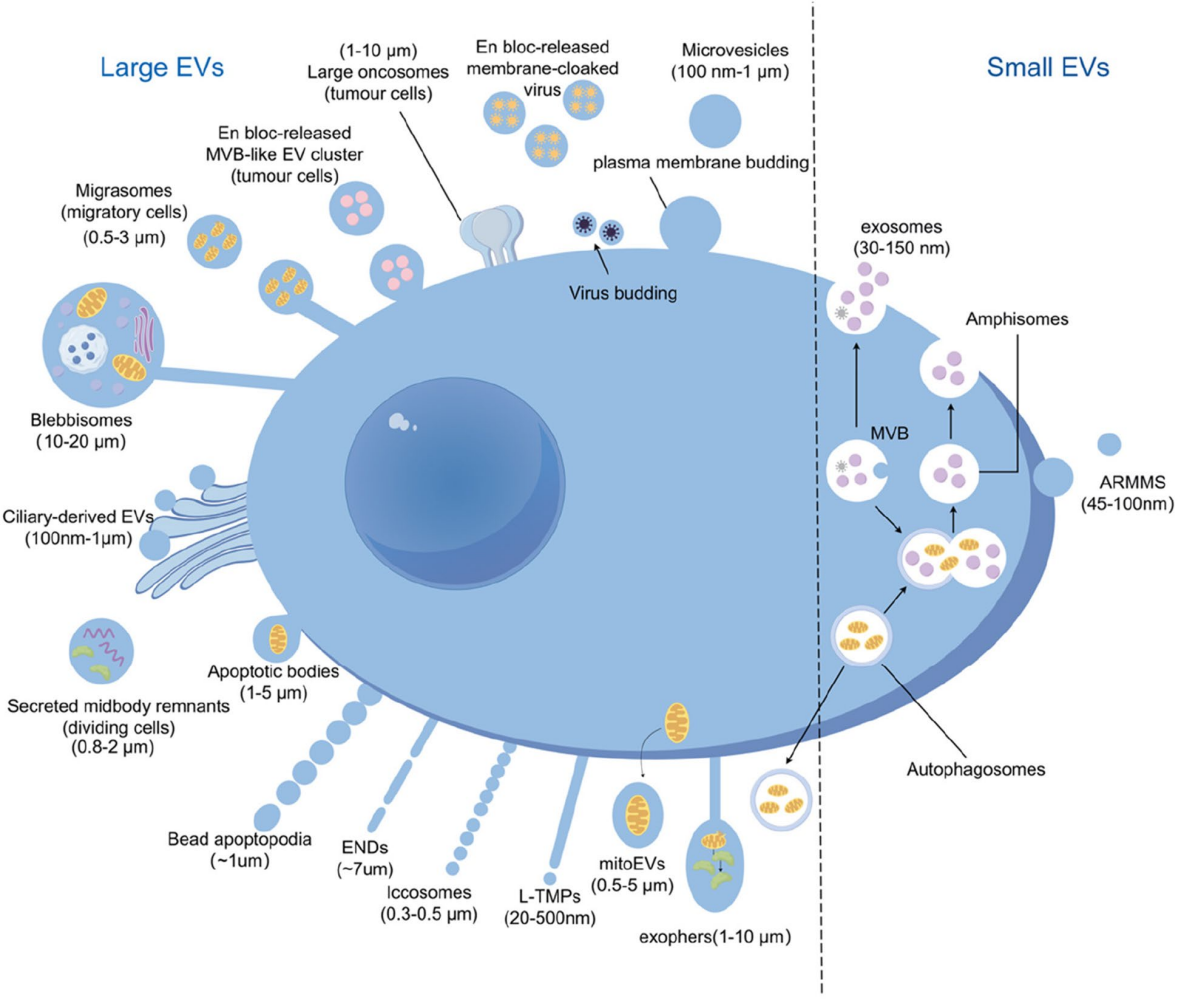
Heterogeneity of classical EVs [13]. The dotted lines indicate the boundaries separating the different categories of EVs based on their size. Large EVs (> 200 nm) are shown on the left, while small EVs (< 200 nm) are shown on the right. Exosomes: Derived from the fusion of MVBs with the plasma membrane. MVs: Formed by the direct outward budding of the plasma membrane. LOs: Released by tumor cells and enriched in various tumor-associated components. EV-like structures of viral origin: Clusters of MVB-like vesicles released in a single step by tumor cells. Apoptotic bodies: Generated during programmed cell death. Migrasomes: Released from the trailing edge of migrating cells. Ciliary ectosomes and secreted midbody remnants: Derived from specific organelles. Virus budding and membrane-cloaked viruses: Illustrate the crosstalk between viral and EV biogenesis pathways. Autophagosome–amphisome–MVB fusion process: Links autophagy with EV generation. ENDS: Elongated membrane-derived structures. LAND-Vs: Vesicles possessing intact lipid bilayers but lacking mitochondria or DNA. Iccosomes: “Beaded dendrite” structures released by follicular dendritic cells (FDCs). mitoEVs: Produced through mitochondrial budding and released into the extracellular space

Large EVs generally refer to a heterogeneous group of membrane-bound vesicles with diameters ≥ 200 nm and a characteristic phospholipid bilayer (Fig. 1). According to the MISEV 2023 guidelines, EV classification should rely primarily on physical characteristics—such as particle size—rather than biogenesis pathways, to reduce confusion arising from variability in isolation techniques. However, whether size alone is sufficient to define EV function or origin remains uncertain and requires further validation [6]. Consequently, most current studies use particle size as the primary classification criterion, supplemented by origin and molecular biomarkers to distinguish specific subtypes. Unless otherwise specified, core functions such as cargo transfer, immune regulation, and microenvironment remodeling are shared by EVs of multiple sizes. The diverse lEV subtypes exhibit distinct characteristics in their size distribution, biogenesis mechanisms, cargo profiles, and functional roles. Table 1 summarizes the key defining features of major lEV subtypes, highlighting their differences from one another and from sEVs.

**Table 1:** Characteristics of Major lEV Subtypes

Subtypes	Size (nm)	Biogenesis	Cell of Origin	Biomarkers	Membrane	Cargo**s**
M**V**s	100–1 000	Plasma membrane budding	Virtually all cell types	ARF6, Annexin V (PS exposure), Annexin A1/A2, integrin (Integrin β1/CD41/CD61), Rho family members, RAB22A	Lipid bilayer	matrix metalloproteinases, proteins, RNAs, metabolites, DNAs
ApoBDs	1000–5000	Apoptotic cell disassembly	Apoptotic cells of various origins	Annexin V (PS exposure), Calreticulin, THBS1, Annexin A1, Histone, Caspase-3, S1PR1	Lipid bilayer	functional organelles, proteins, RNAs, DNAs (Genomic DNA), nuclear fragments, metabolites
LOs	1000–10000	Plasma membrane shedding, Formed under oncogenic stress (e.g., loss of DIAPH3)	Tumor cells	ARF6, caveolin-1, CK18, GAPDH	Lipid bilayer	intact organelles (e.g., mitochondria, ER fragments), oncogenic transcription factors (e.g., MYC), proteins (cytoskeletal proteins), genomic DNA, metabolic enzymes,
Migrasomes	500- 3000	At the termini or junction of retraction fibers; Cholesterol-dependent	Migrating cells	TSPAN4, NDST1, PIGK, CPQ, EOGT, integrins, WGA, lipid markers include Sphingomyelin, Cholesterol, and PI (4,5) P	Lipid bilayer	smaller vesicles, damaged mitochondria, proteins, RNAs, DNAs
Exophers	3500–4000	Stress-induced extrusion, Autophagy-related, LAP-like pathway	Neurons, muscle cell, cardiomyocytes, and other long-lived cells	Huntingtin, Tau protein, LC3, damaged mitochondria, lack Annexin V (PS),	Lipid bilayer	protein aggregates, damaged organelles (e.g., mitochondria), lack nucleic acids
MBRs	800–2000	Cytokinesis	Daughter cells after cytokinesis or stem/progenitor cells	MKLP1/KIF23, BST2/tetherin, RACGAP1, puromycin	Lipid bilayer	ribosomal proteins, RNAs, functional organelles (e.g., ribosomes, endosomes, mitochondria), phosphatidylserine (PS) and phosphatidylethanolamine (PE)
Blebbisomes	10,000–20000	NMIIB-mediated cortical contraction	Tumor cells with NMIIB-dependent blebbing	Dynamic blebbing, contain multiple organelles, bidirectional EVs exchange	Lipid bilayer	functional organelles (e.g., intact mitochondria), high load of immune checkpoints, lack nuclear proteins
Exosomes	30–150	Multivesicular body fusion	Virtually all cell types	Tetraspanins (CD63, CD81, CD9), Alix, and TSG101, Syntenin-1, HSP70, HSP90;	Lipid bilayer	RNAs (e.g., miRNAs, non-coding RNAs), ESCRT-dependent cargos

These characteristics represent *relative enrichment or functional specialization* within lEV subtypes rather than absolute exclusivity

### Microvesicles (MVs)

MVs range from approximately 100 nm to 1 μm in diameter and are generated through outward budding of the plasma membrane [14]. Their biogenesis is regulated by cytoskeletal remodeling and membrane lipid redistribution and involves signaling pathways such as Ca^2^⁺, RhoA/ROCK, and ARF6 [15, 16]. MVs are enriched in phospholipid-binding proteins (e.g., Annexin A1/A2/V), integrins (e.g., CD41/CD61/integrin β1), and membrane-associated regulators such as ARF6 and VAMP3—all of which are rarely detected in sEVs such as exosomes [17–20]. However, these markers are not exclusive to MVs and may also appear in other EV subtypes, reflecting shared biogenetic pathways or challenges in achieving complete subtype-specific isolation. Therefore, assigning functions based solely on these markers remains difficult. Current studies indicate that MV-associated biomarkers vary substantially across tumor types. Examples include the following: Hematological malignancies: CD138 in multiple myeloma and CD30 in lymphoma; Solid tumors: EMMPRIN, EpCAM, HER2/Neu, among others. Notably, breast cancer–derived MVs exhibit high levels of EMMPRIN, which strongly promotes metastatic progression [17]. Nevertheless, these biomarkers are tumor-type–dependent rather than universally MV-specific, and many overlap with markers found in other EV subtypes. Functionally, MVs play critical roles in remodeling the tumor microenvironment, promoting procoagulant activity, and enhancing cancer cell migration [17, 21]. It is important to emphasize that most studies rely on MV-enriched fractions obtained through differential centrifugation rather than fully purified, classical MVs. Consequently, only a subset of vesicles within these preparations display markers characteristic of plasma membrane–derived MVs (e.g., Annexin A1/A2), whereas many co-express canonical sEV markers such as tetraspanins CD63 and CD81. This overlap complicates attempts to classify vesicles based on specific protein markers alone [18, 22]. A specialized MV subtype—apoptotic microvesicles (ApoMVs)—is generated through plasma membrane budding during early apoptosis. Despite their similar size range (100–1000 nm), ApoMVs possess distinct apoptosis-related characteristics, including enrichment of caspase-3, fragmented DNA, and externalized phosphatidylserine (PS). These vesicles are recognized and cleared by macrophages via classical “eat-me” signals (e.g., PS exposure) and can also be internalized by distant target cells through integrin αvβ3-mediated pathways [23, 24]. ApoMVs contribute to physiological processes such as tissue repair, immune modulation, and regulation of stem cell fate [25], but also play roles in pathological conditions including tumor metastasis and osteoporosis [22, 24, 25].

MVs are formed through direct budding from the plasma membrane, a process driven by the redistribution of membrane lipid asymmetry—particularly PS externalization—and contraction of cortical actin [26]. Elevated intracellular Ca^2^⁺ activates scramblases such as TMEM16F, which facilitate PS exposure on the outer leaflet and initiate membrane protrusion [27, 28]. Non-muscle myosin II (NMII) subsequently drives membrane neck constriction and scission, completing vesicle release. Small GTPases (e.g., ARF6, RhoA) and calpain act synergistically to promote rapid MV production, particularly under cellular stress [29, 30]. NMII subsequently drives membrane neck constriction and scission, completing vesicle release. Small GTPases (e.g., ARF6, RhoA) and calpain act synergistically to promote rapid MV production, particularly under cellular stress [31]. ARMMs (Arrestin Domain–Containing Protein 1-Mediated Microvesicles; 45–100 nm) represent a distinct MV subtype generated through direct budding of the plasma membrane mediated by the ARRDC1–TSG101 interaction [32]. ARRDC1 recruits TSG101 via its PSAP motif, initiating membrane budding. ARMMs are enriched in active NOTCH receptors (e.g., NOTCH2) and associated regulators, including the E3 ubiquitin ligase ITCH and the metalloproteinase ADAM10. These vesicles deliver NOTCH receptors to recipient cells, where they undergo γ-secretase–dependent cleavage, enabling non-contact, ligand-independent intercellular communication [33].

### Apoptotic bodies (ApoBDs)

ApoBDs represent a canonical form of lEVs and were the first apoptotic extracellular vesicles (ApoEVs) to be characterized. They typically measure 1–5 μm in diameter and are Annexin V–positive but negative for the platelet marker CD62P [24]. ApoEVs generally comprise three size-overlapping subtypes: ApoBDs (1–5 μm), ApoMVs(100–1000 nm), and apoptotic exosomes (ApoExos; < 200 nm). Dieudé et al. further identified apoptotic exosome-like vesicles released from endothelial cells with diameters of 30–100 nm. Although ApoBDs are commonly defined as 1–5 μm vesicles, their actual size varies considerably depending on the parent cell type and the mechanism of formation. For instance, ApoBDs released by human Jurkat T cells, LIM1215 colon carcinoma cells, and THP-1 monocytic cells may reach diameters of 8–10 μm, whereas numerous bead-like apoptopodia generated by apoptotic THP-1 cells measure < 1 μm [34]. Thus, size alone is insufficient for accurately distinguishing ApoEV subtypes; morphology assessed via electron microscopy and biogenetic pathways must also be considered. First described by Kerr et al. in 1972 as membrane-bound apoptotic debris [35], ApoBDs arise when apoptotic cells undergo orderly disassembly while preserving membrane integrity. This mechanism occurs widely in both physiological and pathological settings [36]. ApoBDs frequently encapsulate intact organelles (e.g., mitochondria, endoplasmic reticulum) [25], nuclear fragments, genomic DNA [24], miRNAs [37], and biologically active proteins such as RNF146 and caspases [38]. Similar to ApoMVs, ApoBDs are engulfed by macrophages through PS-mediated “eat-me” signals and can also be internalized by distant recipient cells via integrin αvβ3-dependent pathways [23, 24]. Histones are highly abundant in ApoBDs derived from stress-induced apoptotic endothelial cells, whereas only trace amounts are found in ApoMVs generated by the same cells. ApoBDs participate in several essential physiological processes, including tissue repair, immune regulation, and modulation of stem cell fate [25]. They are also implicated in multiple pathological conditions, most notably tumor metastasis and osteoporosis [22, 24, 25]. Proteomic analyses reveal that ApoBDs are enriched in ribosomal, cytosolic, nuclear, mitochondrial, and endoplasmic reticulum proteins, whereas ApoExos exhibit higher levels of basement membrane components, extracellular matrix proteins, and lysosomal proteins [39]. ApoExos predominantly express translationally controlled tumor protein (TCTP), tumor susceptibility gene 101 (TSG101) [40], and sphingosine-1-phosphate receptors 1 and 3 (S1PR1 and S1PR3).

ApoBDs are generated through a well-defined, apoptosis-dependent three-step process: membrane budding → membrane contraction → apoptotic body scission [34]. Caspase-3 activation is widely recognized as the central initiating event in ApoBD biogenesis. Caspase-3 cleaves and activates ROCK1, which drives dynamic membrane blebbing and the formation of apoptopodia. Additionally, Caspase-3 regulates Pannexin-1 (PANX1), a caspase-sensitive membrane channel essential for apoptopodia formation; PANX1 cleavage promotes ATP release, which acts as a “find-me” signal to recruit phagocytes. Concurrently, externalized PS functions as an “eat-me” signal facilitating macrophage recognition. PLEXIN B2 has also been identified as a crucial regulator of apoptotic cell disassembly through the formation of beaded apoptopodia, with its function validated in monocyte-based models. Together, these molecules govern the orchestrated structural breakdown of apoptotic cells and the formation of ApoBDs during late-stage apoptosis [24, 41, 42]. Importantly, ApoBDs are not merely passive cellular debris but bioactive messenger vesicles capable of transmitting regulatory signals. For example, ApoBDs containing the E3 ubiquitin ligase RNF146 and miR-328-3p are internalized by mesenchymal stem cells (MSCs) via integrin αvβ3. This interaction activates Wnt/β-catenin signaling, thereby promoting MSC self-renewal and maintaining bone homeostasis. Such findings underscore the dual functional importance of ApoBDs in tissue remodeling and immune regulation [24].

### Large oncosomes (LOs)

LOs, typically 1–10 μm in diameter, were first described by Di Vizio et al. in prostate cancer [43] and have since been identified in multiple tumor types, including breast cancer [22] and glioblastoma [44], highlighting their broad relevance across malignancies. LOs arise under oncogenic stimulation (e.g., MYC, EGFR activation) or metabolic stress such as hypoxia, through extensive membrane budding and scission. They are enriched in oncogenic proteins (e.g., MYC), matrix-degrading enzymes (e.g., MMP2/9), mitochondrial proteins (e.g., TUFM) [45], and oncogenic protein complexes such as ARF6, CK18, and GAPDH. LOs also transport genomic DNA and are capable of reprogramming fibroblasts toward tumor-promoting phenotypes [46]. Functionally, they contribute to the establishment of pre-metastatic niches and play major roles in intercellular signaling within the tumor microenvironment.

LOs share biogenesis features with MVs, as both originate from the plasma membrane. One of the most prominent shared regulators is the small GTPase ARF6, a key driver of MV shedding that is also abundant in LOs. ARF6 is highly expressed in prostate cancer—particularly in metastatic and recurrent lesions—and promotes robust LO release, consistent with aggressive tumor behavior. Loss of DIAPH3, a formin regulated by Rho-family GTPases, further enhances LOs production and is associated with the acquisition of an amoeboid phenotype, a morphological state linked to active LOs shedding [43]. LOs carry a wide array of bioactive cargo, including oncogenic proteins such as AKT and MYC, genomic DNA, and metabolic enzymes including PKM2 [43, 47]. Although the initiating triggers may vary, RhoA activation is widely recognized as a central driver of LOs biogenesis, acting through ROCK- and ERK-dependent pathways to strengthen actin–myosin contractility and promote plasma membrane protrusion [48, 49]. Additionally, in hepatocellular carcinoma (HCC), hypoxia-induced upregulation of cofilin-1 (CFL1) activates the PLD1/AKT pathway, thereby enhancing tumor cell proliferation, migration, and epithelial–mesenchymal transition (EMT) [50]. These findings illustrate how oncogenic signaling and hypoxia jointly fuel the release of LOs, enabling tumor cells to expel toxic intracellular material while disseminating potent pro-tumorigenic signals to neighboring cells.

### Migrasomes

Migrasomes typically measure 0.5–3 μm in diameter and arise at the termini or junctions of retraction fibers formed by migrating cells. They are characterized by the enrichment of specific protein markers, including TSPAN4, NDST1 [51], PIGK, CPQ, EOGT, Integrins [52], WGA [53], Rab35, and Synaptotagmin-1, as well as lipid markers such as sphingomyelin, cholesterol, and PI(4,5)P₂ [54]. Migrasome biogenesis is a multistep process that depends on active cell migration and is tightly regulated by a defined molecular program. The process begins with the nucleation phase. At the leading edge of migrating cells, sphingomyelin synthase 2 (SMS2) forms discrete foci and catalyzes the localized synthesis of sphingomyelin (SM), thereby pre-establishing migrasomes formation sites (MFSs) [55]. This is followed by the maturation phase, during which PI(4,5)P₂ is synthesized at the MFSs by PIP5K1A. PI(4,5)P₂ then recruits the small GTPase Rab35 [56]. Through its interaction with Rab35, integrin α5 is selectively recruited and anchored at these sites, forming a stable connection between the nascent migrasomes and the extracellular matrix [52]. The process concludes with the expansion phase. During this stage, tetraspanins such as TSPAN4 assemble with cholesterol to form tetraspanin-enriched macrodomains (TEMAs) [57], while the calcium sensor Synaptotagmin-1 responds to intracellular calcium signals [58]. Together, these components promote the final expansion and stabilization of the migrasome membrane by enhancing membrane rigidity and supporting the growth of membrane protrusions generated through tube pearling instability induced by membrane tension [56]. Migrasomes encapsulate and transport intracellular damaged components—including dysfunctional mitochondria [59], mRNA [60], and proteins [61]–and subsequently release them into the extracellular space upon rupture of the retraction fibers. Migrasomes play critical roles in diverse biological processes, including intercellular communication, tissue homeostasis, embryonic development, and the pathogenesis, progression, and diagnosis of various diseases [62].

### Exophers

The typical diameter of exophers is 3.5- 4 μm [63]. They were first identified in neurons of *C. elegans* and later observed in *C. elegans* muscle cells and mouse cardiomyocytes [64]. Exopher formation is closely associated with autophagy and represents a conserved cellular strategy—particularly in neurons—for maintaining proteostasis and mitochondrial quality control. The process begins with membrane budding, followed by elongation and constriction. A hallmark of exopherogenesis is the persistent nanotube that connects the forming exopher to its parent cell, enabling ongoing cargo transfer. Ultimately, the exopher is released and subsequently cleared by neighboring phagocytes, functioning as an intercellular waste-disposal mechanism [65]. Exopher biogenesis relies on core autophagy genes such as ATG5 and ATG7, as well as LAP-like pathways involved in membrane encapsulation. Inhibition of autophagy markedly reduces exopher production, whereas efficient clearance by resident macrophages is essential for maintaining tissue homeostasis [63, 64]. In murine hearts, macrophage depletion or impaired phagocytic activity leads to increased inflammation and ventricular dysfunction, underscoring the critical importance of exopher clearance. Furthermore, mitochondrial uncouplers such as FCCP stimulate exopher release, while the MERTK receptor expressed on cardiac macrophages is required for efficient phagocytosis of exophers [64]. Under stress conditions such as proteotoxic stress or mitochondrial damage, exophers encapsulate damaged organelles and protein aggregates (e.g., mutant Huntingtin) into large vesicles that are subsequently “ejected” from the cell and cleared by resident macrophages [66]. Recent studies have revealed non-canonical mechanisms underlying exopher biogenesis. For example, in *C. elegans* neurons, inhibition of early autophagy genes (e.g., ATG-16.2) reduces canonical autophagy but paradoxically enhances exopher secretion. This process is mediated by the WD40 domain of ATG-16.2, indicating that non-canonical autophagy pathways also regulate exopher formation. Functionally, exophers are crucial for detoxification by clearing aggregated polyglutamine proteins and alleviating proteotoxic stress. They may also participate in aging regulation and have been proposed to correlate with lifespan extension [67]. Recent research has identified physiological mechanical force as a key natural signal that triggers exopher generation in vivo. During pregnancy, the maternal body employs a “mechanical force → mechanosensitive channels (PIEZO1/TRP-4) → exopher extrusion” pathway to actively and efficiently eliminate harmful neuronal substances, functioning as a protective quality control mechanism [68].

Emerging evidence also indicates that exopher formation is not solely a cell-autonomous process but is actively regulated by neighboring glial cells. In *C. elegans*, neuronal exopher extrusion triggers phagocytic clearance by surrounding glial-like CEPsh cells. Importantly, this phagocytic activity is not purely degradative; it also stimulates neurons to generate additional exophers via a retrograde signaling cascade. This feedback loop—mediated by VPS-35–dependent retrograde transport and JNK-1 signaling—establishes a coordinated, glia-driven quality control cycle that links debris clearance to sustained extrusion of toxic cellular materials [69]. In response to embryo-derived signals, exophers are also produced as vehicles transporting body wall muscle (BWM)-derived yolk proteins to developing oocytes [70]. Unlike apoptotic vesicles, exophers do not expose PS, indicating a fundamentally distinct biogenetic pathway [71]. Some recent review articles list tau and LC3 as putative exopher cargos, but these remain speculative pending further dedicated studies [72].

### Midbody remnants (MBRs)

MBRs measure approximately 0.8–2 μm in diameter and originate from the midbody structure formed at the conclusion of mitosis, becoming released following cytokinesis [73, 74]. During cell division, the actomyosin contractile ring constricts to draw the plasma membrane inward between the two daughter cells at the site of the spindle midbody. Cytokinesis results in the formation of an intercellular bridge that undergoes coordinated processes, including active constriction, microtubule severing, and membrane remodeling. Concurrently, the bridge recruits the endosomal sorting complex required for transport (ESCRT) machinery, which initiates membrane budding and generates tubules that may be released and internalized by neighboring cells as EVs. Ultimately, the MBR is shed as a distinct subtype of lEVs [65]. MBRs are enriched in translationally active mRNAs and protein synthesis machinery [73, 75]. Patel et al. identified MBRs as a major source of lEVs during the cell cycle. Increasing evidence indicates that MBRs are not merely byproducts of mitosis but may function as intercellular delivery vehicles for mRNA and therapeutic agents, including CRISPR/Cas9 components [74, 75]. Because cancer cells divide rapidly, they generate high quantities of MKLP1-positive lEVs. The MBR-associated protein MKLP1/KIF23 has emerged as a potential pan-cancer marker, with its overexpression reported in glioma, colorectal cancer, gastric cancer, pancreatic ductal adenocarcinoma, lung cancer, breast cancer, and ovarian cancer—frequently correlating with poor prognosis [76]. Importantly, MBRs are not inert cellular debris; they contain bioactive information capable of inducing key biological processes, including cancer cell proliferation, modulation of cell polarity, and even embryonic dorsoventral axis specification [77], establishing them as functional signaling organelles. BST2/tetherin becomes enriched on the surface of MBRs during cytokinesis, anchoring them to the cell surface and preventing premature detachment. Studies show that BST2 knockout results in MBR detachment, accumulation in the extracellular medium, and increased susceptibility to transfer to distant cells [78]. The kinase HIPK2 plays a crucial regulatory role in determining MBR fate. HIPK2 depletion leads to marked intracellular MBR accumulation—comparable to the effects of autophagy inhibition. Mechanistically, HIPK2 promotes MBR clearance not through its classical roles in cytokinesis but by regulating autophagy-mediated degradation: it maintains the levels of the selective autophagy receptors NBR1 and p62/SQSTM1, while sustaining normal autophagic flux. Thus, HIPK2 is a key regulator ensuring timely removal of MBRs via selective autophagy [79].

MBRs are enriched in ribosomal proteins and cell cycle markers such as MKLP1 and RACGAP1 [80]. Classical EV markers (e.g., CD9, CD63, CD81) are also present in midbodies (MBs) and MBRs, contributing to contamination in some preparations labeled as “exosomes.” MBRs carry nucleic acid cargos—including translationally active mRNAs and RNA interference machinery [80, 81]—and are enriched with key proteins involved in cell fate regulation, pluripotency, and oncogenesis (including transcription factors). These mRNAs can be transferred to recipient cells, directly modulating gene expression to promote proliferation, tumorigenesis, and cell fate reprogramming. Additionally, their unique lipid components such as PS and phosphatidylethanolamine (PE) [82, 83] not only support the structural integrity of MBRs but also participate in cell signaling. Together with proteins such as CHMP4B, these lipids further contribute to tumor progression. Collectively, these bioactive molecules establish MBRs as potent vehicles for intercellular communication, playing critical roles in cancer development.

Owing to their enriched cargos and structural characteristics, MBRs hold promise not only as diagnostic biomarkers in cancer but also as therapeutic delivery vehicles, capable of transporting drugs or RNA during mitosis [73, 75]. Research demonstrates that MBRs contain multiple functional components—including ribosomes, endosomes, mitochondria, and RNAi machinery—and exhibit localized protein translation activity. These features firmly support their designation as functional organelles or “mini-cells.”

### Blebbisomes

Blebbisomes, with an average diameter of approximately 10 μm (and reaching up to 20 μm), are the largest functional EVs identified to date. They possess a unique biogenetic mechanism that is distinct from classical EVs. As demonstrated by Jeppesen et al., blebbisome formation is driven by the sudden release of cortical tension mediated by non-muscle myosin IIB (NMIIB) contraction. During cell migration or division, the entire cell surface undergoes rapid and extensive budding, generating large vesicles that encapsulate multiple organelles. Notably, this process occurs independently of the ESCRT complex. Blebbisomes are enriched in functional organelles, including mitochondria, endoplasmic reticulum, and Golgi apparatus, but they notably lack nuclear proteins. Their ability to uptake and subsequently release EVs has earned them the designation of “mobile communication hubs,” highlighting their potential role in tumor immune evasion [45]. Blebbisomes retain dynamic budding activity even after release, sustained by continuous ATP consumption driven by NMIIB contraction. Consequently, the presence of intact mitochondria is critical for maintaining their membrane potential and energy metabolism. These properties confer “pseudo-cell-like” characteristics on blebbisomes, enabling prolonged survival and active interactions within the tumor microenvironment [45].

**Fig. 2:**
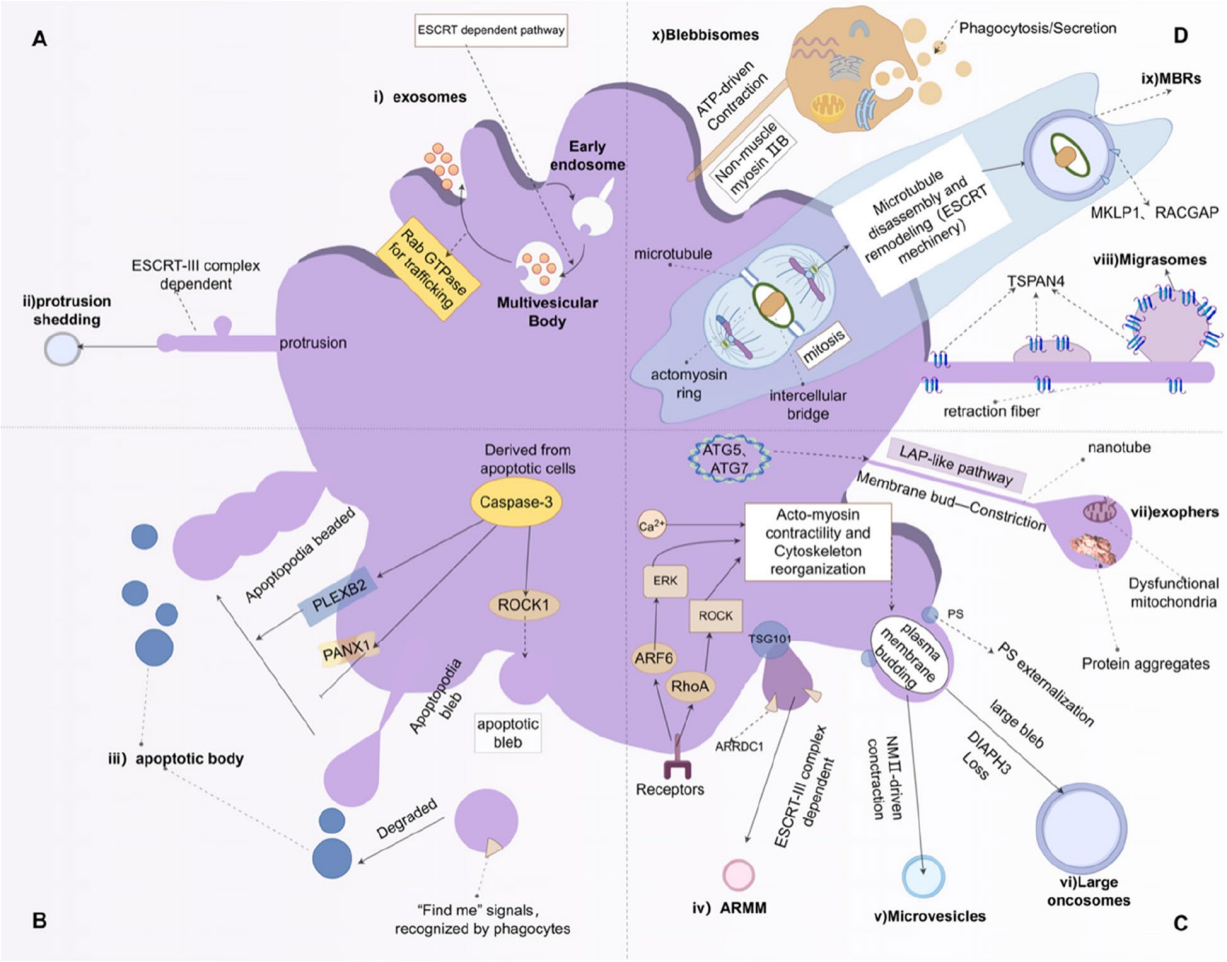
Schematic of EV Biogenesis Pathways [84]. **A** Endosome-Derived Vesicles: i) Formed when early endosomes mature into MVBs via ESCRT-dependent processes. Subsequently, Rab GTPases mediate the fusion of MVBs with the plasma membrane, resulting in the extracellular release of ILVs) as exosomes. ii) Protrusion-Shedding Vesicles: Generated through rupture of membrane protrusions (e.g., microvilli) via the ESCRT-III complex. **B** Apoptosis-Derived Vesicles: iii) ApoBDs: Produced during apoptosis, wherein Caspase-3-mediated cleavage of ROCK1 initiates membrane blebbing and formation of apoptopodia. PANX1, a caspase-regulated channel, and PLEXIN B2 coordinate the development of beaded apoptopodia, which subsequently fragment into multiple ApoBDs. **C** Plasma Membrane-Budding Vesicles: iv) ARMMs: Induced by ARRDC1, which recruits TSG101 from endosomes to the plasma membrane, triggering membrane curvature and vesicle budding. v) MVs: Generated through activation of ARF6/ERK and RhoA/ROCK signaling pathways, which remodel the actin cytoskeleton and promote PS externalization. NMII facilitates membrane scission. vi) LOs: Formed in response to loss of DIAPH3, which destabilizes the cytoskeletal architecture in cancer cells, promoting large-scale membrane budding. vii) Exophers: Stress-induced EVs that encapsulate aggresomes and dysfunctional organelles (e.g., damaged mitochondria). Initially identified in neurons, exophers have also been isolated from other cell types, including cardiomyocytes and muscle cells. Their biogenesis involves core autophagy genes (ATG5/7) and LC3-associated phagocytosis (LAP)-like pathways, including membrane budding, growth via nanotube connections, and phagocytic release. **D** Specialized EVs: viii) Migrasomes: Formed by tetraspanin-enriched membrane microdomains that progressively accumulate on retraction fibers of migrating cells, giving rise to bulbous structures that eventually mature into spherical vesicles. ix) MBRs: Released during cytokinesis as the actomyosin ring constricts the intercellular bridge. ESCRT-mediated tubulation facilitates the formation of secondary EVs from the midbody structure. x) Blebbisomes: Generated via NMIIB-driven membrane blebbing in an ATP-dependent manner. These vesicles are capable of simultaneous secretion and uptake, and notably exclude nuclear content

The biogenesis of lEVs in general involves dynamic membrane remodeling, including cytoskeletal reorganization, membrane budding or shedding, and local regulation of membrane tension. Each lEV subtype exhibits distinct formation mechanisms, reflecting the diverse cellular strategies employed to release vesicles in response to physiological or pathological stimuli. An overview of EV biogenesis pathways is illustrated in Fig. 2.

In summary, lEVs display remarkable structural diversity and mechanistic specificity in their formation. They can be categorized as follows: (1) Membrane remodeling types (MVs, LOs): generated by direct plasma membrane budding under stress conditions; (2) Migration-driven types (Migrasomes): formed at the termini of retraction fibers during cell migration; (3) Autophagy-mediated types (Exophers): involved in the clearance of intracellular damage; and (4) Division-derived types (MBRs): released as residual midbody structures following cytokinesis. These diverse biogenetic pathways enable tumor cells to produce heterogeneous populations of lEVs that are capable of adapting to, manipulating, and remodeling the tumor microenvironment. Among these, blebbisomes—with their retained mitochondria and pseudo-cellular communication capacity—represent a particularly promising frontier for advancing our understanding of tumor metabolic adaptation and immune regulation.

### Several specialized types of EVs

**Fig. 3:**
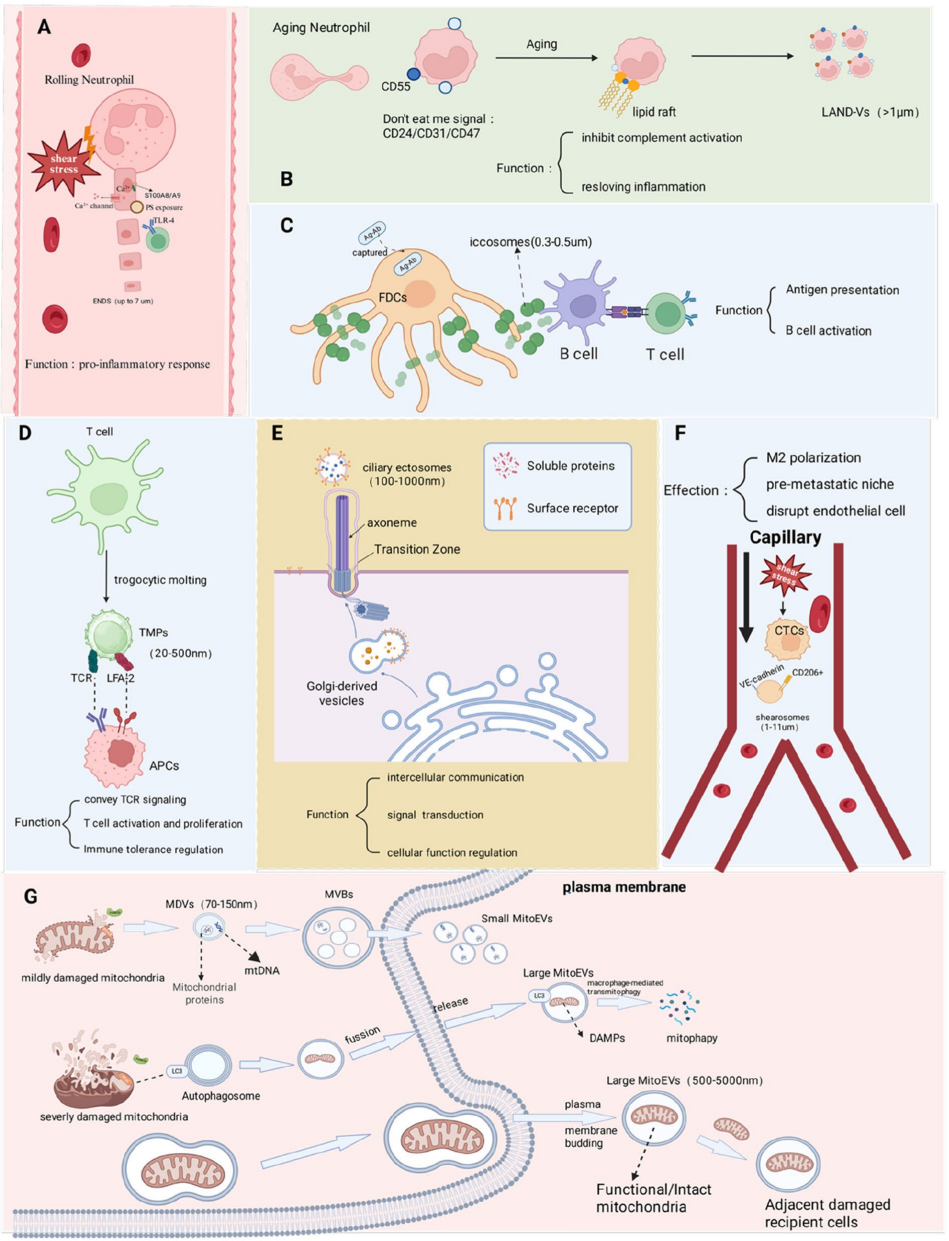
Biogenesis, key molecular regulators, and functional features of major non-canonical lEVs (created with BioRender). **A **ENDS (Elongated Neutrophil-Derived Structures): Generated by neutrophils under blood shear stress, ENDS are enriched in the pro-inflammatory proteins S100A8/A9, whose activity and membrane stability are regulated by Ca^2^⁺. Lacking mitochondria, endoplasmic reticulum (ER), and DNA, ENDS activate the TLR4 pathway, thereby amplifying neutrophil-driven inflammatory responses. **B **LAND-Vs (Large Aging Neutrophil-Derived Vesicles): Formed by aged neutrophils through RhoA–ROCK–dependent plasma membrane budding, LAND-Vs prominently express the “don’t-eat-me” signals CD55, CD24, CD31, and CD47. They lack mitochondria, DNA, apoptotic markers, and MVmarkers such as ARF6/KIF23. Functionally, LAND-Vs inhibit complement activation and evade macrophage clearance, prolonging their persistence during late-stage inflammation and supporting immune homeostasis. **C **Iccosomes (Follicular dendritic cell–derived immune-complex vesicles): Arise from the beaded dendrites of FDCs following the deposition of antigen–antibody complexes. They concentrate immune complexes and facilitate B-cell antigen uptake, thereby contributing to both B-cell and T-cell activation. **D **TMPs (T-cell Microvilli Particles): Released during T-cell activation via trogocytic shedding of microvilli, TMPs contain TCR/CD3, LFA-2/CD2 complexes, and inflammatory cytokines. Large TMPs transfer TCR signals to antigen-presenting cells (APCs), enhance T-cell activation, reprogram lipid metabolism, promote clonal expansion (as indicated by increased Ki-67), and participate in immune tolerance regulation. **E** Cilia-derived EVs: Produced by outward budding from the ciliary membrane, these vesicles are regulated by the axoneme, the selective transition zone, and Golgi-derived vesicles. They contain TSPAN family proteins, ciliary receptors, and PAM, enabling spatially restricted delivery of chemokines and growth factors. These vesicles mediate intercellular communication, signal transduction, developmental patterning, and peptidergic signaling. **F **Shearosomes (Shear stress–induced EVs in circulation): Generated by circulating tumor cells under capillary shear stress, shearosomes express CD206 and VE-cadherin. They contribute to metastatic progression by inducing M2 macrophage polarization, forming pre-metastatic niches, disrupting endothelial integrity, and facilitating tumor-cell extravasation. **G **MitoEVs(Mitochondria-containing EVs). Small MitoEVs (70–150 nm): Originate from the MDV-MVB pathway regulated by PINK1/Parkin, carrying mitochondrial DNA (mtDNA) and proteins. They function in stress signaling, immune modulation, and metabolic adaptation. Large MitoEVs (0.5–5 μm): Formed by direct plasma membrane budding, these vesicles encapsulate entire mitochondria and rely on LC3 and macrophage-mediated transmitophagy. They eliminate severely damaged mitochondria and transfer functional mitochondria to injured cells, restoring metabolic activity. In tumors, MitoEVs may support the metabolic recovery of stromal cells or facilitate tumor adaptation to stress

In recent years, however, an increasing number of non-canonical EVs have been identified and classified under the lEV category. These vesicles exhibit considerable heterogeneity in terms of structure, biogenesis, and function, as summarized in Fig. 3.

ENDS (Elongated Neutrophil-Derived Structures) are elongated membrane protrusions, reaching up to 7 μm, formed by neutrophils under blood shear stress. These structures are enriched in S100A8/A9 and activate the TLR4 pathway, yet they lack mitochondria, endoplasmic reticulum, and DNA, thereby enhancing pro-inflammatory responses. Calcium ions play a dual regulatory role during ENDS formation: they maintain membrane integrity and modulate the activity of S100A8/S100A9 complexes. Specifically, the exclusion of calcium preserves membrane stability, whereas calcium presence activates S100A8/S100A9, amplifying the immune response. Fluctuations in calcium concentration finely tune S100A8/S100A9 function, preventing excessive immune activation and ensuring proper immune modulation [85, 86]. LAND-Vs (Large Aging Neutrophil-Derived Vesicles) are actively produced by aged neutrophils via the RhoA–ROCK signaling pathway. Formed through direct budding of the outer plasma membrane, these vesicles exceed 1 μm in diameter, possess intact lipid bilayers, and lack mitochondria or DNA. LAND-Vs are enriched in CD55 (decay-accelerating factor) and inhibit complement activation by binding C3b/C4b, thereby evading macrophage clearance. Additionally, the co-expression of “don’t-eat-me” signals, including CD24, CD31, and CD47, prolongs their persistence during late-stage inflammation, supporting immune homeostasis and extending neutrophil-mediated immunological functions. LAND-Vs display minimal expression of mitochondrial markers (Tom20, Tim23) and MV markers (ARF6, KIF23), and lack apoptotic markers such as cleaved Caspase-3/7. They are comprehensively identified using flow cytometry (CD55⁺/CD47⁺/CD66b⁺), imaging (large size > 1 μm), and electron microscopy (absence of internal organelles) [85–87]. Iccosomes, measuring 0.3–0.5 μm in diameter, originate from the “beaded dendrites” of FDCs following immune complex (Ag–Ab) deposition. These vesicles play a critical role in antigen transport and B-cell activation by concentrating antigens, which are subsequently internalized by B cells [88]. Protrusion Shedding Vesicles are generated from the rupture of membrane protrusions, including microvilli, filopodia, and cilia, through mechanisms dependent on ESCRT-III components such as CHMP4B and Vps4B. Their formation is often induced by cellular stress or membrane-damaging factors, including hyaluronic acid and cholesterol, and is particularly prevalent in immune cells. These vesicles facilitate the transfer of membrane proteins and promote immune activation. Unlike exosomes, which rely on the multivesicular endosome pathway, protrusion-shedding vesicles depend on cytoskeleton-driven membrane deformation and direct mechanical forces. Because these protrusions function as cellular “sensors” and “interactors,” vesicles released from these sites are likely specialized for localized and directional cell-to-cell communication. Although these EVs share overlapping physical characteristics and biochemical markers with other subtypes, they can be distinguished by a combination of specific protein markers and an understanding of their unique biogenetic pathways. For instance, Prominin-1 specifically targets microvilli and cilia. EVs enriched in CD9 or CD81 but lacking CD63 are more likely derived from the plasma membrane, including protrusions, whereas those enriched in CD63 with minimal CD9 are typically of endosomal origin [89]. Notably, T cell–derived TMPs (T cell Microvilli Particles) belong to this category. Kim et al. (2018) reported that the diameter of large TMPs (L-TMPs) is approximately 500–20 nm, whereas small, exosome-sized TMPs (S-TMPs) measure roughly 40–20 nm [33, 84, 90, 91]. TMPs are formed via microvilli “trogocytic molting” during T cell activation and are enriched in TCR/CD3, LFA-2/CD2 complexes, and inflammatory cytokines. Recent studies in 2023 revealed that TMPs not only facilitate immune communication but also reactivate TCR expression, reprogram lipid metabolism, enhance Ki-67 expression, and promote T cell clonal expansion—highlighting TMPs as critical drivers of T cell proliferation. Among lEV subtypes, TMPs are notable for their capacity for both bidirectional signaling and self-renewal, emphasizing their unique regulatory role at the immunological synapse [90, 91]. Furthermore, the ciliary membrane generates cilia-derived ectosomes (100–1000 nm) via outward budding, a tightly regulated process that ensures selective incorporation of membrane and soluble proteins. The axoneme, the microtubule-based core of the cilium, interacts with the ciliary membrane to facilitate proper vesicle formation. Simultaneously, the transition zone acts as a selective diffusion barrier, controlling protein entry and exit to precisely sort cargo into the vesicles. Golgi-derived vesicles deliver essential proteins and enzymes to the ciliary membrane, supporting vesicle biogenesis. During peptidergic signaling, Peptidylglycine α-amidating monooxygenase (PAM) plays a key role in ciliary ectosomes, activating peptides involved in gamete interactions and other intercellular signaling processes. These mechanisms collectively enable ciliary ectosomes to perform essential functions in intercellular communication, signal transduction, and cellular regulation. While similar in size to s, MVs arise via direct plasma membrane budding and generally lack the precise internal sorting mechanisms of ciliary ectosomes. Consequently, MVs incorporate a broader range of cellular components and are not as specialized for peptidergic signaling [92]. Amphiectosomes (diameter > 350–500 nm) contain both LC3B-positive intraluminal vesicles (ILVs) from autophagosomes and CD63-positive ILVs from MVBs, encapsulating multiple ILV subtypes. This structure represents a “multi-subtype EV co-release” pathway, linking autophagy and classical EV biogenesis [93]. Additional lEVs include shearosomes, measuring 1–11 μm in diameter. These vesicles are enriched in protein markers related to immune regulation and endothelial function, including CD206, a marker of M2 macrophages, and VE-cadherin, which is critical for endothelial barrier integrity. Shearosomes contribute to pre-metastatic niche formation, a crucial step in tumor metastasis. They disrupt endothelial barrier integrity, facilitating tumor cell extravasation, while also activating immune cells—particularly monocytes and macrophages—promoting their polarization toward the M2 (tumor-promoting) phenotype, thereby supporting tumor growth and metastasis. Shearosomes have been shown to impair endothelial cell integrity in both 2D and 3D models, highlighting their essential role in metastatic progression. Collectively, these findings suggest that shearosomes enable circulating tumor cells to traverse capillary beds efficiently and enhance metastatic tumor formation through coordinated effects on endothelial and immune cell functions [94]. Other endothelial-derived lEVs expose PS to mediate mitochondrial elimination and monitor distal vascular damage [95]. MitoEVs, lEVs ranging from 0.5–5 μm, are enriched in intact mitochondria, mitochondrial DNA (mtDNA), and metabolic enzymes. They play critical roles in intercellular metabolic communication and tumor regulation [26, 27, 84]. Large MitoEVs, a recently identified subtype, are formed by direct mitochondrial budding from the plasma membrane, independent of Drp1 or endoplasmic reticulum involvement. These vesicles are distinct from traditional mitochondrial-derived vesicles (MDVs, 70–150 nm) [96] or mitovesicles, representing a new class of mitochondrial-origin EVs [97–99]. In contrast, small MitoEVs generally arise via the MDV–MVB pathway, regulated by the PINK1/Parkin mitochondrial quality-control axis. These vesicles typically carry mildly damaged mitochondrial components, including mitochondrial fragments, mtDNA, and metabolic enzymes [100, 101], and are primarily involved in local signaling, innate immune regulation, metabolic adaptation, and intercellular stress communication. Large MitoEVs, however, form through plasma membrane outward budding and can encapsulate entire mitochondria, either polarized or depolarized, thereby conferring dual biological functions: removing damaged organelles and supplying functional mitochondria to recipient cells. Firstly, large MitoEVs function as a critical quality-control mechanism, selectively removing severely damaged mitochondria that cannot be processed through the MDV–MVB pathway. This clearance process relies on LC3 and is ultimately executed via macrophage-mediated transmitophagy, engaging phagocytic receptors to prevent the accumulation of inflammatory DAMPs, such as mtDNA, thereby maintaining cellular homeostasis [102]. Secondly, large MitoEVs can deliver functional mitochondria to recipient cells, restoring oxidative phosphorylation, ATP production, and overall metabolic status in neighboring injured cells—effects that are particularly important for tissue repair following ischemic or inflammatory injury. Thus, while small MitoEVs primarily act as mediators of intercellular signaling, large MitoEVs serve a dual role: as “disposal units” for severely damaged mitochondria and as “metabolic support vehicles” facilitating intercellular mitochondrial transfer. This dual functionality underscores their greater therapeutic potential in conditions involving mitochondrial dysfunction. In the tumor microenvironment, MitoEV-mediated transfer of functional mitochondria or mtDNA may allow tumor cells to escape metabolic quiescence, promoting proliferation and survival, while simultaneously restoring metabolic activity in damaged stromal cells [101]. However, the lack of specific isolation methods for MitoEVs poses a challenge in purifying mitochondrial-origin EV subpopulations from heterogeneous EV pools. The precise mechanisms underlying mitochondrial packaging, targeting specificity, and functional regulation of MitoEVs in cancer remain largely undefined and require further investigation. Recent studies have also demonstrated that M2 macrophage-derived MitoEVs can transfer mitochondrial components to induce adipocyte-to-myofibroblast transition, mediating fibrosis via the TGF-β/PAI-1 pathway, which may contribute to tumor-associated stromal remodeling [103]. Collectively, these findings substantially expand our understanding of the functional diversity of lEVs. The lEV family exhibits extensive structural and biogenetic complexity, with cargo composition directly shaping their roles in tumor progression. Future studies employing multi-omics and single-vesicle approaches are essential to define subtype-specific features and functional heterogeneity, ultimately advancing the clinical translation of lEVs as diagnostic biomarkers and therapeutic targets.

In contrast, exosomes (30–150 nm) —the classical sEVs — are formed via the endosomal pathway. In this process, early endosomes mature into MVBs, and the ILVs within MVBs are released as exosomes upon fusion with the plasma membrane. They are characterized by tetraspanins (CD9, CD63, CD81) and ESCRT-associated proteins (Alix, TSG101, Syntenin-1), reflecting their endosomal origin. Unlike lEVs, exosomes lack Annexin A1/A2, confirming their distinct biogenetic pathway from plasma membrane–budding vesicles [19, 104, 105].

## Molecular cargo and functions of lEVs in tumor progression

Large EVs serve as central mediators of intercellular communication within the tumor microenvironment (TME). While many of the cargo types and basic functions described below—such as the transport of nucleic acids, lipids, and specific proteins—are general hallmarks of EVs, largely elucidated through studies of sEVs, the larger size of lEVs enables the packaging of unique cargo complexes, including fragments of organelles and multiple vesicles, as well as a pronounced quantitative enrichment of specific molecules. This expanded cargo capacity can amplify their functional impact compared with smaller EVs. The biologically active cargo of lEVs—including proteins, nucleic acids, lipids, and metabolites—plays pivotal roles in regulating signaling pathways, driving metabolic reprogramming, and orchestrating immune responses. Through these mechanisms, lEVs actively contribute to tumor initiation, progression, and metastasis, often exerting effects that are complementary to, yet distinct from, those of sEVs.

### Protein cargo and functional roles

#### MVs

MVs are enriched with a diverse array of biologically significant molecular cargos. Among their protein cargos, small GTPases such as ARF6, RhoA, and RAB22A play pivotal roles in regulating MV formation and release in cancers, including breast and prostate cancer. Notably, ARF6 is highly enriched in MVs and governs multiple downstream signaling pathways. The protease MT1-MMP is selectively incorporated into MVs via the ARF6–VAMP3 pathway, promoting tumor cell invasion [29]. Elevated expression of the mucin MUC1 in tumor cells, such as HeLa cells, enhances MV release [106]. Cytoskeleton-associated proteins, including Fascin, ERK, MLCK, and MLC, are regulated by ARF6/RhoA signaling and facilitate the constriction and scission of MV necks [29]. Under hypoxic conditions in breast cancer, RAB22A mediates the localization of transglutaminase-2 (TGM2) to MV budding sites [107].

With respect to nucleic acid cargos, MVs derived from prostate cancer contain large chromosomal DNA fragments (up to 2 Mb), reflecting the genomic alterations of the parent tumor. Furthermore, the ARF6–GRP1 complex mediates the sorting of Exportin-5–pre-miRNA into MVs, representing a miRNA loading mechanism distinct from exosomes [108]. Under oxidative stress, hnRNPA2B1, together with miRNAs, is selectively incorporated into MVs in a Caveolin-1 phosphorylation-dependent manner [109]. Lipids, including cholesterol and phospholipids, contribute to MV membrane stability and biogenesis, and depletion of cholesterol significantly suppresses MV release [110]. In addition, during the differentiation of AML-M5 leukemia cells, MVs are released carrying mitochondria, underscoring their role as versatile intercellular messengers capable of transferring both macromolecular and organelle cargos.

#### ApoBDs

ApoBDs exhibit multifaceted functions in maintaining tissue homeostasis and regulating immune responses, distinguishing them from other ApoEV subtypes, such as ApoMVs and ApoExos. During tissue regeneration, ApoBDs are efficiently cleared by macrophages through recognition of surface “eat-me” signals, including PS, Calreticulin, Annexin A1, and thrombospondin 1 (THBS1). This rapid phagocytic removal contributes to anti-inflammatory resolution and promotes tissue repair [111, 112]. Beyond their clearance, ApoBDs carry bioactive signaling molecules capable of modulating stem and progenitor cell behavior. For instance, they influence mesenchymal stem cells (MSCs) via the αvβ3–RNF146–miR-328-3p/Wnt–β-catenin axis, thereby regulating bone homeostasis [24]. Wang et al. [113] reported that ApoBDs derived from MSCs induce apoptosis in multiple myeloma cells, resulting in symptomatic improvement. Mechanistically, ApoBDs trigger a calcium influx, promoting translocation of Fas from the cytoplasm to the cell membrane. Simultaneously, they present Fas ligand (FasL), activating the Fas/FasL pathway and generating reactive oxygen species (ROS), ultimately inducing cell death. Polymorphonuclear cell-derived ApoBDs can activate plasmacytoid dendritic cells (pDCs), stimulating the secretion of interferon-alpha (IFN-α) via vesicle-contained DNA [114]. Multimodal studies further demonstrate their roles in angiogenesis and wound healing [112, 115]. ApoEVs derived from stem cells are internalized by endothelial cells and enhance angiogenesis by transferring the mitochondrial factor TUFM and promoting autophagy through a TFEB-dependent mechanism [116]. In an endometrial injury model, ApoBDs incorporated into a hydrogel promoted tissue repair and reduced fibrosis by enhancing cellular proliferation, angiogenesis, and modulating inflammation [117].

ApoBDs also display cell type-specific functions in bone remodeling and tumor-related processes. Derived from distinct osteoclast precursors, such as preosteoclasts and mature osteoclasts, ApoBDs inherit unique molecular profiles that dictate their therapeutic potential. For example, ApoBDs from mature osteoclasts enhance osteogenic differentiation via activation of RANKL reverse signaling, whereas those from preosteoclasts promote vascularization through PDGF-BB and the PI3K/AKT pathway [118–120]. These vesicles also carry specific long non-coding RNAs (lncRNA), contributing to their regulatory roles in bone metabolism and tumor microenvironment modulation [118]. Importantly, ApoBDs exhibit superior osteogenic activity compared to other EVs, including MVs and exosomes, highlighting their potential in treating bone defects and primary or metastatic bone tumors [118, 120].

ApoBDs derived from macrophages have been shown to enhance adipogenesis and suppress osteogenesis in mesenchymal stem cells by delivering miR-155 to target the SMAD2 signaling pathway [121]. During apoptosis, ApoBDs can encapsulate a variety of autoantigens, including nucleosomal DNA, nuclear ribonucleoproteins, and SSA/Ro52, through processes involving cytoskeletal reorganization and JNK-mediated apoptotic signaling [122–124]. This inheritance of parent cell-derived autoantigens highlights the potential role of ApoBDs in autoimmune pathogenesis and supports their theoretical utility as vaccine candidates for autoimmune diseases. Additionally, ApoBDs carrying tumor-derived components from parent cells can serve as an antigen source for cancer vaccines [125].

Despite their promise, the clinical translation of ApoBDs faces multiple challenges. Their high heterogeneity is influenced by factors such as parental cell characteristics, biogenesis mechanisms, and preparation protocols, while the precise mechanisms underlying molecular inheritance during formation remain elusive. Furthermore, limitations including lack of standardized production, immunogenicity and safety concerns, and limited sources collectively hinder clinical application. To overcome these challenges, future research should focus on: elucidating the in vivo behavior and molecular mechanisms of endogenous ApoBDs; establishing standardized production and characterization systems; optimizing safety through engineering strategies; and developing technical platforms to investigate complex mechanisms, thereby systematically advancing clinical translation.

In contrast, ApoMVs, although also derived from apoptotic cells, do not induce sterile inflammation, emphasizing that the pro- or anti-inflammatory effects of apoptotic vesicles are source- and subtype-dependent. ApoExos, on the other hand, display distinct immunogenic features: in comparative studies, only ApoExos were confirmed to act as damage-associated molecular patterns (DAMPs) in mice, triggering immune activation [126]. A notable advantage of ApoBDs over ApoExos is their non-immunogenic profile: enriched with proteins specialized in RNA processing and targeted delivery, ApoBDs do not elicit autoantibody production or transplant rejection, whereas ApoExos provoke immunogenic responses and are predominantly associated with proteasomal degradation and ligase functions. Consequently, ApoBDs represent an immunologically quiescent, pro-resolving EV subtype, ApoExos act as immunogenic alarm signals, and ApoMVs occupy an intermediate, largely non-immunogenic role.

#### LOs

The abundance of LOs is associated with increased tumor aggressiveness [127]. A summary of the principal molecular functions of LOs across different cancer types is provided in Table 2.

**Table 2:** Roles of specific molecules in LOs within different cancers

Cargos	Cancer/cancer model	Pathophysiological process involved
EGFRvIII	Cancer (type unspecified)	Mediates horizontal transmission of oncogenic signals and drives oncogenic transformation in recipient cells
14–3-3 and β-catenin	Multiple cancer models	Stabilize β-catenin levels; Co-induce LOs formation/secretion; Activate Wnt signaling; Induce cytoskeletal rearrangement; Enhance cell migration
ATP6V1G1	Glioblastoma	Promote tumor metastasis; Acidify the microenvironment
VAPA	HCC (subpopulation with high bone-metastatic potential)	Drive osteoclastogenesis and bone-tropic metastasis
αV-integrin	HCC, Prostate Cancer	HCC: Acts as an LO marker and 'sorter' for VAPA; Mediate LO adhesion to recipient cells Prostate Cancer: it’s enrichment level significantly correlates with high Gleason scores and lymph node metastasis; Drive tumor invasiveness
MMP2/MMP9	Tumor models (unspecified)	Delivered to the peritumoral stroma; Degrade extracellular matrix components; Facilitate tumor cell invasion and metastasis
Metabolic Enzymes (GAPDH, LDHB, MDH, GOT1, GLS)	Prostate and Bladder Cancer models	Enriched in LOs (GLS is exclusive to LOs); Participate in intercellular metabolic reprogramming
Cytoskeletal/Motility-related Proteins (Actin, Tubulin, Cofilin, Profilin)	Prostate and Bladder Cancer models	Enriched in LOs; Associated with cell motility and morphology
Membrane Trafficking & Signaling Proteins (Annexins A1/A2/A6, G-proteins)	Prostate and Bladder Cancer models	Involved in membrane trafficking and signal transduction
CK18	Prostate Cancer	Serves as a characteristic marker for identifying tumor-derived LOs; its presence is closely associated with PCa progression
PCa-specific Palmitoylated Proteins (STEAP1, STEAP2, ABCC4)	Prostate Cancer	Significantly enriched in LOs; Provide highly specific biomarker candidates for non-invasive diagnosis
Transcription Factors (HOXA7, HOXA10, POU3F2)	Glioblastoma	Translocate to the nucleus to modulate gene expression and cellular fate (upon internalization); Contribute to pro-tumorigenic signaling
Mitochondria-associated Proteins (ATP5A1, ATP5B, HSPD1, LRPPRC, SLC25A5, IMMT)	Prostate Cancer	Enriched in LOs derived from prostate cancer
uPAR and eEF1γ	Prostate Cancer models	Enriched in LOs; contribute to cancer progression
miR-1227	Tumor models (unspecified)	LOs carry threefold more miR-1227 than exosomes; its overexpression enhances the promotion of cancer-associated fibroblast migration

Research demonstrates that LOs mediate the intercellular transfer of the oncogenic protein EGFRvIII to recipient tumor cells that originally lack this receptor. This horizontal transmission facilitates the dissemination of tumor-promoting signals and drives oncogenic transformation in recipient cells [128]. Di Vizio et al. (2009) first identified that oncosomes contain both 14–3-3 and β-catenin. Building on this, Dovrat et al. (2014) elucidated the mechanism by which 14–3-3 and β-catenin drive tumor progression across multiple cancer models. Their work demonstrated that these proteins not only cooperate intracellularly to stabilize β-catenin levels but also co-induce the formation and secretion of oncosomes enriched with both molecules. It is noteworthy that this regulatory mechanism is not exclusive to oncosomes, as sEVs have also been reported to contain and transmit similar signaling cargo. Once internalized by neighboring cells, these oncosomes can activate the Wnt signaling pathway in a Wnt ligand-independent manner. This activation induces cytoskeletal rearrangement and enhances cell migration, thereby facilitating the intercellular transmission of oncogenic signals [127, 129, 130]. In glioblastoma, the membrane-bound V-ATPase subunit ATP6V1G1 can modulate vesicle fusion with target cells and acidify the microenvironment, thereby promoting tumor metastasis [131]. Zhang et al. (2022) reported that the VAPA protein is specifically enriched in LOs shed by a HCC subpopulation with high bone-metastatic potential, dictating their organotropic behavior. Their study demonstrated that VAPA, via its Major Sperm Protein (MSP) domain, directly interacts with the LO marker αV-integrin, facilitating the sorting and surface localization of VAPA on LOs. This establishes VAPA as a critical cargo protein for LO-induced osteoclastogenesis, playing a pivotal role in bone-tropic metastasis of HCC. Moreover, αV-integrin serves not only as a canonical LO marker and a "sorter" recruiting VAPA into LOs but may also mediate LO adhesion to recipient cells [7]. LOs significantly influence stromal and tumor cell behavior within the tumor microenvironment [43, 132]. They upregulate metastasis-associated factors, including BDNF, CXCL12, and osteopontin, in stromal cells, while also enhancing the migration of tumor and endothelial cells. Notably, circulating LOs from metastatic mice promote the migration of normal endothelial cells, underscoring their role in tumor-stroma communication [43]. Additionally, LOs deliver proteolytic enzymes such as MMP2/MMP9 [47] to the peritumoral stroma, where they degrade extracellular matrix (ECM) components, thereby facilitating tumor invasion and metastasis [133]. In both prostate and bladder cancer models, the protein cargo profile of LOs is fundamentally distinct from that of sEVs. Unlike sEVs, which are enriched in classical exosomal markers, LOs are uniquely enriched in a broad spectrum of metabolic enzymes and cytoskeleton-associated proteins. LOs contain metabolic enzymes such as GAPDH, LDHB, MDH, GOT1, and GLS, with GLS being exclusive to LOs and absent in sEVs. As GLS catalyzes the conversion of glutamine to glutamate and plays a key role in cancer cell glutaminolysis, its specific enrichment suggests that LOs may contribute to intercellular metabolic reprogramming. Concurrently, LOs are highly enriched in cytoskeletal and motility-related proteins, including Actin, Tubulin, Cofilin, and Profilin, supporting their role in cell migration and structural remodeling.

Furthermore, LOs also carry membrane trafficking and signaling proteins, including Annexins A1, A2, A6 and G-proteins [134]. The specific molecular cargo of LOs plays a critical role in the progression and diagnosis of prostate cancer (PCa). For example, cytokeratin 18 (CK18) serves as a characteristic marker for tumor-derived LOs, with its presence in both tissues and plasma closely correlating with PCa progression. Notably, αV-integrin, enriched on the LO surface, shows a significant association with high Gleason scores and lymph node metastasis, underscoring its central role in driving tumor invasiveness and metastasis, and highlighting its potential as a prognostic indicator and therapeutic target. Proteomic analyses have further identified significant enrichment of PCa-specific palmitoylated proteins, including STEAP1, STEAP2, and ABCC4, within LOs, providing highly specific biomarker candidates for non-invasive diagnosis of PCa. Collectively, these findings underscore the important functions of LOs and their cargo in malignant progression and highlight their potential for clinical diagnosis and precision therapy [43, 135, 136]. Transcription factors, such as HOXA7, HOXA10, and POU3F2, are packaged within LOs. Upon uptake by recipient cells, these transcription factors can translocate to the nucleus, modulating gene expression and cellular fate, thereby contributing to pro-tumorigenic signaling, particularly in glioblastoma progression [43, 131]. Mitochondria-associated proteins are also enriched in prostate cancer-derived LOs, including ATP5A1, ATP5B (mitochondrial ATP synthase subunits), HSPD1 (heat shock protein 60), LRPPRC (mitochondrial RNA processing factor), SLC25A5 (mitochondrial carrier protein), and IMMT (mitochondrial inner membrane protein) [137]. Studies in prostate cancer models revealed that LOs are enriched in specific pro-tumorigenic factors, such as urokinase-type plasminogen activator receptor (uPAR) and eukaryotic elongation factor 1 gamma (eEF1γ), which contribute to cancer progression [138].

Although analyses of LOs remain preliminary, comparative miRNA profiling has revealed distinct enrichment patterns for specific miRNA species compared to smaller EVs [132]. In functional assays, LOs derived from miR-1227-overexpressing tumor cells promoted cancer-associated fibroblast migration more effectively than exosomes, carrying approximately threefold more miR-1227 due to their larger size.

#### Migrasomes

Migrasomes carry a diverse array of biomolecules and organelles, including proteins, nucleic acids, lipids, and specific cellular structures. In terms of protein cargo, migrasomes are enriched not only in intrinsic membrane proteins such as tetraspanins (e.g., TSPAN4) and integrins but also in a wide range of signaling molecules, including chemokines (e.g., CXCL12), cytokines, and growth factors (e.g., VEGFA) [139–141]. Vesicles originating from the secretory pathway and their associated secretory proteins, as well as cytosolic and other organellar proteins (e.g., the late endosomal GTPase Rab7), are also present within migrasomes [51, 142]. Regarding nucleic acids, migrasomes selectively enrich full-length mRNAs (e.g., PTEN mRNA) and various small RNA species. Under conditions of mild mitochondrial stress, they encapsulate damaged mitochondria and thus contain mitochondrial DNA [59, 143]. Their characteristic morphological features also include numerous small vesicles derived from the secretory pathway [51, 141]. With respect to lipid composition, migrasomes are enriched in key lipids such as sphingomyelin, cholesterol, and PI(4,5)P₂, which provide critical structural support while also participating in signaling processes [56, 144]. Through these diverse cargos, migrasomes perform several essential physiological functions.

First, migrasomes function as carriers that mediate the transfer of materials and information between cells. After being engulfed by neighboring cells, the mRNAs contained in migrasomes (e.g., PTEN mRNA) can enter the cytoplasm of recipient cells and be translated into functional proteins, thereby altering the recipient cells’ behavior and enabling lateral transfer of biomolecules [143]. Second, migrasomes play a central role in maintaining cellular homeostasis, particularly by mediating mitocytosis. Through this process, damaged mitochondria are selectively packaged into migrasomes and expelled from the cell. This mechanism is essential for preserving mitochondrial membrane potential and respiratory function in highly migratory cells (e.g., circulating neutrophils), and it represents a mitochondria quality-control process tightly coordinated with cell migration [59]. Finally, migrasomes act as efficient platforms for the targeted delivery of signaling molecules. They concentrate chemokines and growth factors at specific spatial locations—such as the embryonic shield cavity during development or along angiogenic pathways—and release these factors through localized exocytosis or leakage. In doing so, migrasomes establish local signaling gradients that provide precise spatial and chemical cues for embryonic patterning, angiogenesis, and immune regulation [139, 140, 142]. In recent years, migrasomes—organelles tightly associated with cell migration—have gained increasing attention for their roles in tumor biology. Given that their formation depends on cell migration, highly migratory tumor cells are considered an important source of migrasomes. Studies indicate that migrasomes contribute not only to tumor invasion and metastasis but also modulate the tumor microenvironment through the bioactive molecules they carry. For example, TSPAN4, a key structural protein of migrasomes, has been shown to correlate significantly with tumor-associated macrophages [145], suggesting a potential role for migrasomes in immune regulation. Growing evidence highlights the possible clinical significance of migrasomes in cancer. By integrating pan-cancer omics data with single-cell sequencing, one study found that the expression of migrasome-related genes is strongly associated with poor patient prognosis and may represent promising targets for cancer immunotherapy [146]. In gliomas, including glioblastoma multiforme and lower-grade glioma, TSPAN4 expression demonstrates notable tumor specificity. Moreover, its differential expression across immune subtypes suggests that this gene may promote tumor progression through multiple molecular mechanisms [147]. Migrasomes, functioning as information carriers generated during cell migration, exert their biological effects through the diverse bioactive molecules they contain. Studies have shown that immune cells, such as dendritic cells, can use migrasomes to deliver cytoplasmic components (e.g., antigens and chemokines) to adjacent cells [148]. Migrasomes are enriched with mRNAs and proteins that can be laterally transferred to recipient cells; notably, these mRNAs can be translated into functional proteins within recipient cells, directly modulating their functional states [143]. This migrasome-mediated information transfer is distinct and highly specific, as it strictly depends on the cell migration process. Accordingly, migrasomes are thought to primarily convey migration-related information—such as key cues for cell polarization and directional guidance—coordinating directed movement and spatial organization at the multicellular level.

#### Exophers

Exophers represent a distinct mode of intercellular communication, characterized by a non-classical clearance mechanism involving coelomocytes rather than traditional apoptotic pathways, which enables long-distance transfer of cellular material [70]. Their physiological importance is highlighted by essential roles in development and tissue homeostasis: in *C. elegans*, exophers released from body wall muscles support embryonic growth—a function that is conserved in murine models [70]. Likewise, in the murine heart, exophers are vital for cardiomyocyte maintenance, mediating mitochondrial quality control and proteostasis by exporting damaged organelles for degradation by resident macrophages. Despite these recognized biological functions, the mechanisms underlying exopher biogenesis and their potential contributions to cancer remain poorly understood. Given their capacity to alleviate proteotoxic stress, remove dysfunctional mitochondria, and promote growth, it is plausible that dysregulated exopher production could substantially influence cancer cell metabolism and proliferation [64, 70].

#### MBRs

A recent study in colorectal cancer revealed that MBRs are enriched with a broad array of proteins, including midbody structural components (KIF23/MKLP1, RACGAP1, CEP55, AURKB, PLK1), RNA granule–associated proteins (FUS, TARDBP, IGF2BP1/2), splicing factors and ribonucleoproteins (HNRNPs, SRSF, SF3B), mitochondrial proteins (VDAC1/2, TOMM22), histones (HIST1H1C, HIST2H3A), translation initiation factors (the EIF family), and endoplasmic reticulum–associated proteins (HSPA5, CANX, ERP44). These enriched proteins participate predominantly in pathways related to RNA transport, spliceosome activity, endoplasmic reticulum protein processing, and the tricarboxylic acid (TCA) cycle. Moreover, colorectal cancer–derived MBRs also contain several proteins associated with cancer progression, including MSH2/MSH6 (DNA mismatch repair proteins), STAT1, TGM2, MDK, and PRKACA, suggesting that MBRs may exert pro-tumorigenic or regulatory influences within the tumor microenvironment [149].

#### Multi-omics–identified lEV-enriched cargos

A recent study employed high-resolution density gradient centrifugation to isolate lEVs from patients with non-small cell lung cancer (NSCLC), revealing two functionally distinct subtypes: low-density lEVs (ld-lEVs) and high-density lEVs (hd-lEVs). Among these, ld-lEVs are predominantly enriched in membrane-associated proteins and play a central role in immune regulation. Notably, these vesicles specifically carry PD-L1, and their plasma abundance has been identified as an independent predictive biomarker for response to anti–PD-1 immunotherapy in patients exhibiting low or negative PD-L1 expression in tumor tissues. This subtype-specific enrichment—where PD-L1 is almost exclusively present in ld-lEVs and nearly absent in hd-lEVs—explains why PD-L1 often cannot be detected when analyzing total EVs in tissue-negative patients, whereas targeted isolation of ld-lEVs can effectively uncover this key immunosuppressive pathway. Moreover, ld-lEVs are enriched with αVβ5 integrin (ITGAV + ITGB5) and tumor-associated membrane proteins such as EpCAM and CD44, which may collectively facilitate tumor–microenvironment interactions and influence metastatic behavior and adhesion dynamics. In contrast, hd-lEVs are enriched in cytoplasmic, nuclear, and metabolism-related proteins, with biological functions skewed toward cell adhesion and migration. Their cargo includes small GTPases involved in intracellular trafficking (e.g., Rab5, Rab7, Rab10), cytoskeletal components (e.g., Actin, Tubulin, Vimentin), and metabolic enzymes (e.g., GAPDH, PKM, ENO1), suggesting a potential role in reshaping the metabolic landscape of recipient cells. Importantly, hd-lEVs also contain DNA damage repair proteins (e.g., XRCC5, XRCC6, PARP1) and nuclear proteins (e.g., Histones H3/H4, Lamin A/C), clearly indicating the presence of nuclear material. This likely originates from nuclear membrane budding or nuclear leakage under cellular stress, implying a mechanism by which genomic instability may be transmitted from tumor cells to recipient cells. Collectively, ld-lEVs and hd-lEVs derived from NSCLC exhibit distinct molecular signatures and biological functions, and their refined subtyping provides valuable insights into tumor progression mechanisms and the development of innovative liquid biopsy approaches [150]. Additionally, recent analysis of the lEV surface proteome by Alin Rai et al. (2021) [129] has further illuminated the marked differences in cargo composition between lEVs and sEVs. lEVs are enriched with proteins and RNAs originating from intracellular organelles such as mitochondria and the endoplasmic reticulum, suggesting that their formation may result from large-scale cytoplasmic blebbing rather than the highly regulated endosomal vesicle–generation pathway. In contrast, sEVs function as a “rich repository” of plasma membrane and endomembrane system–related proteins, displaying enrichment in membrane-associated proteins and canonical exosomal markers, consistent with their derivation from MVBs or plasma membrane microdomains. Nevertheless, more than 50% of proteins and a large number of transcripts are shared between lEVs and sEVs, indicating the presence of a core, non-selective cargo packaging mechanism common to both vesicle types.

lEVs encapsulate a wide spectrum of functional proteins essential to tumor biology, enabling intercellular signaling and reprogramming of the tumor microenvironment. Although many of these proteins are also present in sEVs, their abundance or specific combinatorial patterns within lEVs may confer distinct functional properties.

### Nucleic acid cargo and functional role

lEVs are enriched with a diverse array of nucleic acids—including miRNAs, lncRNAs, mRNAs, and DNA fragments—that collectively regulate gene expression in recipient cells and shape tumor phenotypes. 1) miRNA: lEVs carry oncogenic miRNAs such as miR-21 [150] and miR155 [150, 151], which are commonly upregulated in multiple tumor types. These miRNAs promote tumor cell proliferation and invasion primarily by repressing tumor suppressor genes, including PTEN. 2) mRNA: lEVs mediate the transfer of mRNAs encoding oncogenic signaling molecules, such as mutant KRAS and MYC. Upon translation in recipient cells, these transcripts drive clonal expansion and malignant transformation [152]. 3) lncRNAs and circRNAs: These non-coding RNAs act as regulators of miRNA activity, thereby facilitating tumor cell proliferation, invasion, and immune evasion [14]. 4) Mitochondria-associated mRNA: In prostate cancer, lEV-associated mRNAs are enriched for the mitochondrial gene MTCO1 (cytochrome c oxidase subunit 1), suggesting that lEVs may participate in remodeling energy metabolism externally [137]. 5) Transcription factors: Transcription factors such as HOXA7, HOXA10, and POU3F2 are also packaged within lEVs [43]. After uptake, these proteins translocate to the nucleus of recipient cells, modulating gene expression and cell fate. This mechanism has notable pro-tumorigenic effects, particularly in the progression of glioblastoma [43]. The stability and tumor specificity of these nucleic acid cargos highlight the promising potential of lEVs as biomarkers for liquid biopsy–based cancer detection and monitoring.

### Lipid and metabolite cargo and function

Although traditional EV research has focused predominantly on proteins and RNAs, emerging evidence highlights the critical roles of EV lipids in tumor metabolism and immune modulation [47].

#### PS

Exposed on the surface of lEVs, PS facilitates macrophage-mediated phagocytosis by engaging receptors such as MFGE8 and Gas6, thereby contributing to immune tolerance and promoting tumor immune evasion [153].

#### Cholesterol

Highly enriched in lEV membranes, cholesterol stabilizes vesicle structure, prolongs circulation half-life, and enhances delivery efficiency. It also plays a pivotal role in vesicle budding, with high cholesterol content representing a hallmark of EV formation—particularly for plasma membrane–derived vesicles [154]. Regulation of a cholesterol-centered “lipid axis” is essential for lEV biogenesis; cholesterol accumulation not only promotes lEV generation but may also influence membrane rigidity, targeting specificity, and cargo delivery efficiency. Notably, inhibition of MEK signaling or cholesterol biosynthesis reduces lEV release and sensitizes cancer cells to chemotherapy, underscoring the therapeutic potential of targeting lipid composition in lEVs [155].

#### Sphingolipids

Sphingolipids are essential for maintaining EV structural integrity and supporting intercellular communication. For instance, sphingosine-1-phosphate (S1P) within EVs can induce T-cell exhaustion, whereas glycolipids such as ganglioside GD3 inhibit T-cell receptor signaling [156].

#### Metabolites

Metabolites including glucose, lactate, and glutamine can modulate the energy metabolism of recipient cells through “metabolic coupling,” playing a crucial role in sustaining metabolic adaptation in hypoxic tumor microenvironments [157].

#### Ketone bodies

Recent studies show that β-hydroxybutyrate (β-HB), a key ketone body metabolite, functions not only as an alternative energy source but also actively promotes the biogenesis of MDVs. β-HB induces mitochondrial oxidative stress and mild membrane depolarization, driving the selective packaging of damaged proteins and oxidized lipids into vesicles released extracellularly. This process involves classical Rab GTPase signaling and DRP1-mediated mitochondrial fission. These findings broaden our understanding of the diverse origins of lEVs and suggest that specific metabolic states—such as elevated ketone body levels—may regulate EV release, cargo composition, and functional potential across both small and large vesicles. This metabolic modulation of lEV dynamics offers novel insights for targeted metabolic interventions [158]. Importantly, several characteristics originally described for sEVs, including metabolite-mediated intercellular signaling, are also shared by lEVs and may be amplified by their larger cargo capacity.

Taken together, these observations indicate that EVs of all sizes form a complex lipid- and metabolite-based “metabolic communication network” that underpins tumor metabolic reprogramming, immune evasion, and microenvironmental homeostasis. Within this shared system, lEVs represent an enriched, size-enabled subset with enhanced functional capacity. As a sophisticated and highly dynamic EV population, lEVs transmit and regulate multidimensional information within the tumor microenvironment through their protein, nucleic acid, lipid, and metabolite cargos. These cargos are integral to multiple stages of tumor progression and provide an important foundation for non-invasive diagnostics, disease monitoring, and precision therapeutics.

## Biological and pathophysiological roles of lEVs in tumor progression

Large EVs exert multilayered regulatory effects throughout key stages of tumor initiation, progression, and metastasis. By transporting diverse signaling molecules and metabolic components, they contribute to local invasion, pre-metastatic niche formation, immune evasion, and therapeutic resistance, thereby emerging as pivotal modulators of the tumor ecosystem.

### Local invasion and metastasis

Ovarian cancer cells with elevated EFNB expression release lEVs upon contact with peritoneal mesothelial cells. These lEVs carry EFNB and activate EphB signaling, inducing mesenchymal transition of mesothelial cells and promoting lymphangiogenesis, thereby facilitating peritoneal metastasis. Importantly, lEV release is strongly dependent on direct cell–cell contact, suggesting that their influence is largely confined to the local tumor microenvironment. This EFNB–EphB communication axis may represent a novel therapeutic strategy for preventing peritoneal dissemination [15]. LOs further enhance invasive capacity by activating the AKT–MYC pathway via αV-integrin. This activation upregulates inflammatory and angiogenic mediators—including IL-6, CXCL12, and BDNF—in cancer-associated fibroblasts (CAFs), normal-associated fibroblasts (NAFs), and endothelial cells, collectively increasing cancer cell invasiveness and adhesion [43]. Additionally, lEVs enriched with matrix-degrading enzymes such as MMP2 and MMP9 directly degrade the basement membrane and extracellular matrix (ECM), thereby promoting tumor cell motility and local infiltration [134].

### Pre-metastatic niche formation in distant organs

The integrin subtype profile on lEVs dictates metastatic organotropism. lEVs enriched with α6β4 and α6β1 integrins preferentially home to the lungs, whereas those carrying αvβ5 integrins target the liver [159]. Upon arrival, these vesicles activate endothelial and stromal cells in the target organs, inducing inflammatory cytokine secretion, increasing vascular permeability, and recruiting myeloid cells—ultimately establishing a pre-metastatic niche conducive to tumor colonization and growth. LOs enriched with VAPA can activate osteoblast precursors, thereby promoting bone-tropic metastatic targeting [7]. At the primary tumor site, cancer stem cells (CSCs) generate LOs through the NRF2–ATG9B axis. ATG9B scramblase activity loads AnnexinA1 onto the surface of these LOs. Annexin A1⁺ LOs then deliver IL-33 to myeloid precursor cells expressing the FPR2 receptor, driving their differentiation into Arg1⁺ CD206⁺ FcεRIα⁺ immunosuppressive macrophages. This process establishes a TGF-β–rich immunosuppressive microenvironment that supports CSC self-renewal, enhances local tumor progression, and provides mechanistic insights into pre-metastatic niche formation at distant sites [160]. MBRs may also serve as active contributors to pre-metastatic niche development by systemically delivering oncogenic proteins and RNAs to distant organs, thereby remodeling the local microenvironment to support metastatic colonization. Shearosomes enhance interactions between endothelial and immune cells, creating a milieu favorable to tumor cell adhesion and colonization at secondary sites [161]. Mitochondria-enriched lEVs (mito-lEVs) also play emerging and significant roles in pre-metastatic niche formation. For example, mito-lEVs released by M2 macrophages carry intact mitochondria and metabolites that reprogram the metabolic state of target tissues. These vesicles promote adipocyte-to-myofibroblast transition, activate TGF-β and PAI-1 signaling pathways, and remodel the extracellular matrix—collectively contributing to fibrosis and facilitating metastatic colonization [103].

### Tumor immune suppression

Extensive research has established the classical immune regulatory mechanisms of sEVs, particularly exosomes, in mediating immune evasion, suppression, and activation. Key findings include:

1. Immune suppression: sEVs carrying PD-L1 bind to PD-1 on T cells, inhibiting their activity and inducing functional exhaustion, thereby facilitating tumor immune escape [155, 162, 163]. Regulatory T cell (Treg)-derived sEVs expressing CTLA-4 interact with CD80/CD86 on dendritic cells (DCs), impairing antigen presentation and blocking T cell activation. Immunosuppressive miRNAs (e.g., miR-21, miR-155, miR-29a) within EVs modulate signaling pathways such as STAT3, SOCS1, and PTEN in immune cells, suppressing T cell and NK cell cytotoxic functions [164]. Combined targeting of STAT3 (e.g., with selective inhibitors) and PTEN (e.g., via gene therapy approaches) may synergistically enhance the efficacy of immunotherapy. Recent studies (2025) further reveal that EV-associated PD-L1 not only antagonizes T cell activation but also induces DNA damage and perturbs lipid metabolism within T cells, promoting senescence and exhaustion and thereby compromising antitumor immunity. Blocking EV release or therapeutically targeting these metabolic pathways in T cells can partially reverse such dysfunction [165]. Tumor-derived lEVs carrying inhibitory ligands including PD-L1, PD-L2, B7-H3, and VISTA may exert simultaneous suppressive effects on multiple immune cell populations, further enhancing tumor immune evasion [9]. In addition, EVs expressing FASL or TRAIL can induce T cell apoptosis via interactions with FAS, DR4, and DR5 receptors.
2. Inhibition of DC maturation and antigen presentation: Tumor-derived EVs downregulate MHC I/II and CD80/CD86 expression on dendritic cells, impairing DC maturation and disrupting subsequent T cell priming [9]. EV-associated soluble mediators such as TGF-β and surface molecules including HLA-E can reprogram DCs toward a tolerogenic phenotype, further diminishing their antigen-presenting capacity [166]. Although several pathways have been implicated, the underlying regulatory mechanisms remain incompletely understood and continue to be an active area of investigation.
3. Delivery of immunosuppressive molecules and activation of downstream signaling pathways: Blebbisome-type EVs are enriched in ectoenzymes such as CD73, which catalyze adenosine production in the TME. Adenosine is a potent immunosuppressive metabolite that inhibits T cell and NK cell activity [9]. Furthermore, triple-negative breast cancer (TNBC)-derived microparticles can transport immunosuppressive cytokines such as IL-6, activating the gp130/STAT3 pathway in macrophages to promote M2 polarization; activation of the AKT/mTOR pathway further reinforces this immunosuppressive state [166]. Collectively, these mechanisms support tumor immune evasion while maintaining a chronically inflamed yet immunosuppressed microenvironment.
4. Macrophage polarization: Tumor stem cell–derived LOs carry interleukin-33 (IL-33), which drives monocytic myeloid precursors toward an M2-like immunosuppressive macrophage phenotype, thereby contributing to the establishment of a tumor-permissive, immune escape microenvironment [160].

Despite increasing recognition of their biological significance, studies on lEV-mediated immune regulation remain relatively limited. Available evidence indicates that lEVs enriched in PD-L1 can capture and inactivate T cells within lymph nodes, thereby facilitating systemic immune evasion. The biogenesis of lEVs is closely linked to TGF-β1–induced intracellular Ca^2^⁺ influx and calpain protease activation [16]. lEVs carrying PD-L1, miR-155, or adenosine suppress dendritic cell (DC) expression of MHC I/II and co-stimulatory molecules, thereby impairing antigen presentation and attenuating T cell priming. Adenosine accumulation generated by these vesicles further exacerbates both DC dysfunction and T cell exhaustion [167]. Treg-derived lEVs containing TGF-β and miR-155 can recruit myeloid-derived suppressor cells (MDSCs), which inhibit effector T cell activity, expand Treg populations, and secrete IL-10 and TGF-β—collectively promoting immune tolerance and tumor progression [168]. Certain lEV populations also exert profound effects on macrophage biology. For example, shearosomes, enriched with immunomodulatory proteins, are actively internalized by immune cells and induce monocyte polarization toward a pro-tumorigenic M2 macrophage phenotype. These vesicles disrupt endothelial barriers and enhance tumor dissemination [161]. Similarly, melanosomes (size-consistent with lEVs) secreted by melanoma cells can be transferred sequentially to keratinocytes, fibroblasts, and ultimately macrophages, inducing diverse polarization states—ranging from pro-tumorigenic to pro-inflammatory. These lEV-like melanosomes further promote angiogenesis and distant metastasis via the AKT1/mTOR/VEGF axis, and their abundance correlates with melanoma aggressiveness and poor immunotherapy response [169]. Recent work demonstrates that TGF-β stimulation drives cervical cancer cells to secrete medium/large EVs (> 130 nm) enriched in CD39 and CD73, which generate immunosuppressive adenosine in the TME, thereby suppressing T cell activation and enabling immune escape [164]. In HCC, lEVs carrying PKM2 reprogram the TME by polarizing macrophages toward the M2 phenotype [159]. lEV formation is further regulated by a TGF-β1–activated type I receptor–MEK–ERK1/2 axis, which phosphorylates SREBP2 and upregulates DHCR7, increasing EV cholesterol content and establishing a regulatory “cholesterol axis” governing lEV biogenesis [155]. TGF-β also reshapes EV protein composition—such as by increasing MMP9—thereby promoting tumor cell migration and drug resistance. Inhibiting MEK or cholesterol biosynthesis reduces lEV release and restores tumor chemosensitivity. Large EVs additionally contribute to chemotherapy resistance. In bladder cancer, upregulation of GSTO1 promotes lEV-mediated cisplatin efflux, reducing DNA adduct formation and conferring drug resistance. Pharmacological inhibition of lEV release reverses this effect, identifying a promising therapeutic strategy [161].

**Fig. 4:**
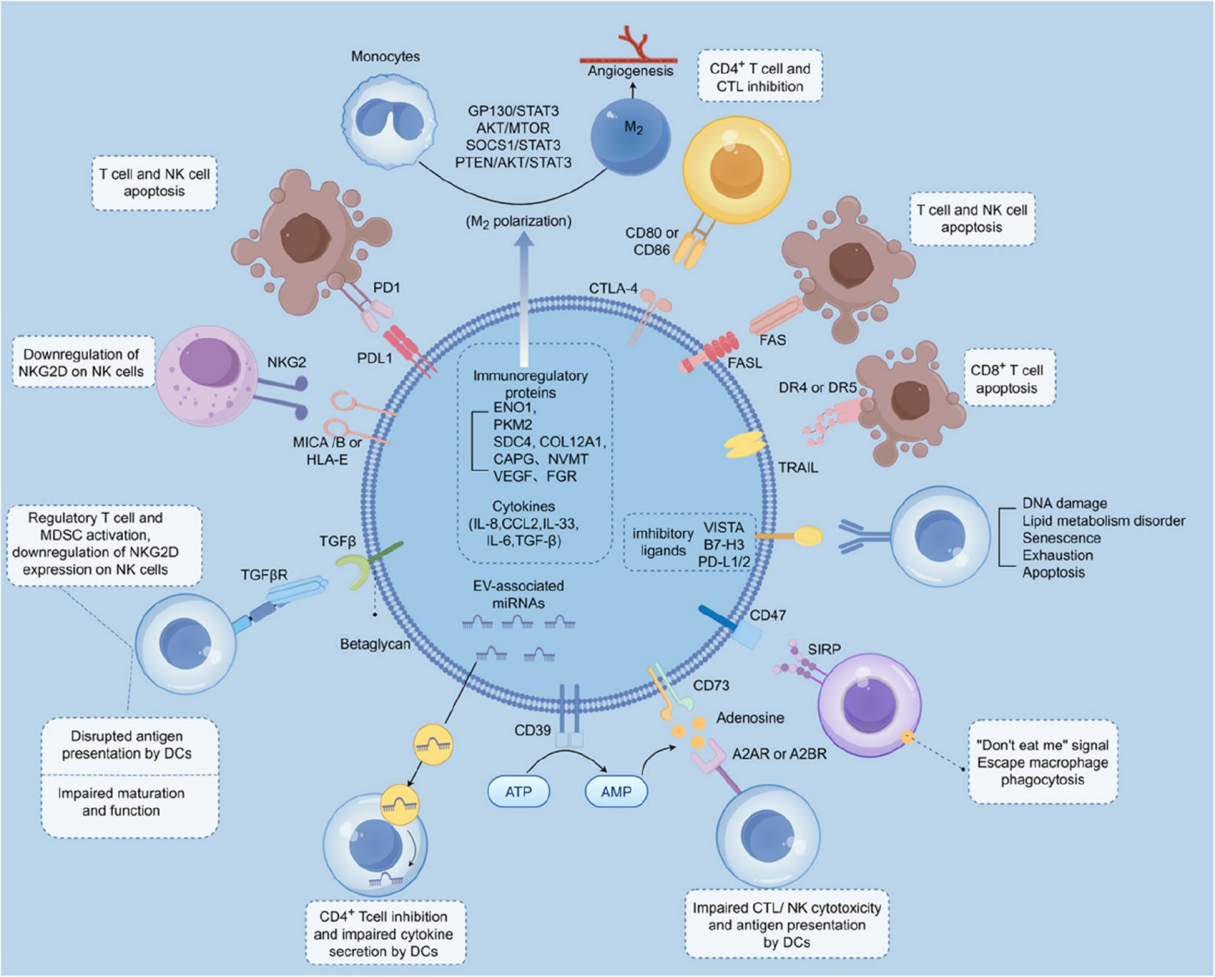
Immune regulatory functions of EVs [13]. **a** lEVs transport cytokines (e.g., IL-6, IL-8, IL-33, CCL12, TGF-β) and immunoregulatory proteins (e.g., ENO1, PKM2, SDC4), activating signaling pathways such as gp130/STAT3, which drive macrophage polarization toward an M2 immunosuppressive phenotype. This polarization promotes angiogenesis and enhances tumor invasion and metastatic dissemination. **b** Immunomodulatory surface molecules on EVs—including immune checkpoint ligands such as PD-L1 and CTLA-4, as well as apoptosis-inducing ligands FASL and TRAIL—engage their corresponding receptors on T cells and natural killer (NK) cells, suppressing their cytotoxic activity or initiating apoptosis. **c** lEVs bearing multiple inhibitory ligands, such as PD-L1, PD-L2, B7-H3, and VISTA, can concurrently modulate diverse immune cell subsets. Notably, PD-L1 not only inhibits T cell responses but also induces DNA damage and lipid metabolic dysfunction, thereby promoting T cell senescence and exhaustion. **d** CD47 on lEV surfaces interacts with its receptor on T cells and phagocytes, delivering a “don’t eat me” signal that enables immune evasion. **e** Ectonucleotidases CD39 and CD73 convert extracellular ATP into adenosine, a potent immunosuppressive metabolite that impairs cytotoxic T lymphocyte (CTL) activity and diminishes antigen-presenting capacity in dendritic cells (DCs). **f **Regulatory T cell–derived EVs transport miRNAs that inhibit CD4⁺ T cell activation (e.g., miR-155, miR-21) or modulate cytokine production by acting on DCs (e.g., miR-150-5p, miR-142-3p). **g** Surface-bound TGF-β on EVs interacts with betaglycan, activating Tregs and myeloid-derived suppressor cells (MDSCs) while downregulating the activating receptor NKG2 on NK cells. **h** EVs presenting NKG2 ligands—such as MICA/B and HLA-E—can further downregulate NKG2 on NK cells and impair DC maturation and function

Beyond oncology, lEV-like vesicles also demonstrate significant immunomodulatory activity in non-cancer systems. ApoBDs derived from mesenchymal stem cell (MSC) apoptosis can robustly induce M2 macrophage polarization and enhance phagocytosis, highlighting their stability and therapeutic delivery potential in vivo [170, 171]. In HIV research, lEVs enriched in miR-155 increase viral infectivity and disrupt CD4⁺/CD8⁺ ratios. Targeting DCIR in mouse models reduces viral burden and restores immune homeostasis, suggesting broader implications for lEVs in inflammatory regulation and tumorigenesis [172]. The immunomodulatory functions of EVs are summarized in Fig. 4.

### Environmental factor-induced lEVs mediate EMT and tumor metastasis

Growing evidence indicates that environmental pollutants not only cause direct genetic damage but also rewire intercellular communication to promote cancer development. 1,4-Dioxane (1,4-D)—a volatile organic contaminant commonly present in industrial wastewater and consumer products—activates the Nrf2 signaling pathway in human bronchial epithelial cells, thereby inducing the secretion of lEVs enriched with oncogenic cargo. Nrf2 activation selectively upregulates metastasis-associated genes (e.g., SDC4, COL12A1, CAPG, NNMT), which are efficiently packaged into lEVs.

These lEVs exhibit potent oncogenic activity: they induce EMT in recipient cells and significantly enhance cellular migration, invasion, and proliferation. Notably, Nrf2 deletion markedly reduces SDC4 expression, lEV cargo loading, and downstream EV uptake, thereby diminishing these pro-oncogenic effects. This mechanistic framework represents the first defined “pollutant → signaling pathway → lEV biogenesis → cargo loading → EMT” axis, highlighting lEVs as key intermediaries linking environmental carcinogens with tumor initiation and progression [173].

### lEVs mediate perineural invasion via glial cell activation

Perineural invasion (PNI) is a major driver of the aggressiveness and poor prognosis of pancreatic ductal adenocarcinoma (PDAC). Recent studies demonstrate that PDAC-derived lEVs, enriched with interleukin-8 (IL-8) and CCL2, activate Schwann cells and induce their transition into a pro-invasive, tumor-supportive phenotype. These activated Schwann cells create a “chemoattractant corridor” that promotes tumor cell migration along nerve bundles. PDAC lEVs enhance Schwann cell chemokine secretion, adhesion capacity, and extracellular matrix remodeling, thereby markedly increasing PDAC neurotropism in co-culture and transwell migration assays. This establishes a self-amplifying positive feedback loop between tumor cells and peripheral nerves. These findings highlight lEVs as critical signaling mediators in tumor–nerve crosstalk and essential contributors to PNI pathogenesis [174].

Beyond their mechanistic role in PNI, EVs also show strong potential as liquid biopsy biomarkers for early PDAC detection. A large-scale proteomic analysis using EVtrap technology identified several plasma EV proteins—such as PDCD6IP, SERPINA12, and RUVBL2—that were significantly elevated in PDAC patients compared with individuals with benign pancreatic lesions. Additional markers linked to metastasis (e.g., PSMB4, ANKAR) and prognosis (e.g., CD55, RALB) were also identified. Notably, a 7-protein EV signature achieved 89% accuracy in distinguishing PDAC from benign controls, offering promising opportunities for non-invasive early screening and personalized therapeutic stratification [175].

### Roles of lEVs in metabolic diseases

Large EVs also contribute to the pathogenesis of metabolic diseases. In mouse models, injection of lEVs or sEVs from insulin-resistant (IR) and non-IR individuals demonstrated that IR-lEVs inhibit insulin signaling in adipose and liver tissues, induce adipocyte hypertrophy, and upregulate lipogenic gene expression. Mechanistically, IR-lEVs are enriched in active protein tyrosine phosphatase 1B (PTP1B), whereas IR-sEVs contain protein phosphatase 2 A (PP2A), which synergistically disrupt insulin signaling. Inhibition of these enzymes reverses these effects, identifying novel therapeutic targets for metabolic dysfunction [176]. In cancer metabolism and drug resistance, lEVs perform multiple roles. They deliver pro-angiogenic factors such as vascular endothelial growth factor (VEGF) and fibroblast growth factor (FGF), promoting endothelial proliferation and vascularization at metastatic sites [14, 105, 169]. Large EVs also transfer drug efflux transporters (e.g., ABCC1), anti-apoptotic microRNAs (e.g., miR-21, miR-222), and metabolic enzymes (e.g., ENO1, PKM2), collectively enhancing chemoresistance [153, 161]. Under chemotherapy-induced stress, certain lEVs transport mitochondrial components, including mtDNA and ATP5A1, to mediate metabolic reprogramming [153]. A study on porcine seminal plasma sEVs found that these vesicles possess surface structures capable of resisting DNA oxidative damage caused by hydrogen peroxide (H₂O₂), whereas lEVs lack this protective capacity. This suggests size-dependent functional diversity among EV subtypes and highlights their differential therapeutic potential [177, 178].

In summary, lEVs contribute to tumor progression through diverse mechanisms, including the direct delivery of oncogenic signals, remodeling of pre-metastatic niches, immune suppression, promotion of angiogenesis, and adaptation of the host microenvironment. These multifaceted roles underscore lEVs as critical regulators within the tumor ecosystem.

## Blebbisomes: a novel extracellular vesicle in the tumor microenvironment

Blebbisomes represent a groundbreaking advancement in EV research. With complex structures and multifunctional capabilities, they may redefine current paradigms of intercellular communication. Compared to conventional EVs (e.g., exosomes and MVs), blebbisomes exhibit distinct differences in biogenesis, composition, and biological function, particularly in tumor immune regulation, signal integration, and metabolic reprogramming.

### Structure and characteristics of blebbisomes

Blebbisomes are currently regarded as the most functionally promising subtype of lEVs due to their unique features, including their mode of biogenesis (NMIIB-mediated membrane contraction), large size (10–20 μm), organelle content (e.g., functional mitochondria, Golgi apparatus), and prolonged dynamic activity (sustained blebbing for over 72 h). Key characteristics include: 1) Organelle enrichment [9, 10]: Blebbisomes contain functional organelles such as mitochondria, endoplasmic reticulum, Golgi apparatus, and MVBs, but notably lack nuclei. This composition renders them similar to “enucleated cells” with metabolic and biosynthetic potential. The enrichment of intact mitochondria suggests that blebbisomes may reprogram the metabolism of recipient cells through the transfer of mitochondrial components (e.g., mitochondrial DNA and proteins), thereby influencing tumor metastasis. 2) Dynamic activity [9, 10]: Following their release, blebbisomes retain active “blebbing” behavior for dozens of hours, maintaining membrane dynamics and structural plasticity. 3) Bidirectional communication capability [9, 10]: Blebbisomes can internalize exogenous EVs (e.g., by phagocytosing surrounding exosomes) and actively secrete new EVs (30–1000 nm, including exosomes and MVs), forming a cascading intercellular signaling network. This dual capability has earned blebbisomes the designation of “motherships” of the EV world. 4) Immune regulatory molecule enrichment [9, 10]: Blebbisomes are densely packed with immunosuppressive molecules (e.g., PD-L1, PD-L2, B7-H3, VISTA, HLA-E, CD47) and components of the adenosine pathway (e.g., CD73), forming a robust, multilayered immunosuppressive signaling platform.

### Roles of blebbisomes in tumor immune evasion

Emerging evidence suggests that tumor-derived blebbisomes, enriched with immunosuppressive molecules, play critical roles in facilitating immune escape through multiple mechanisms [10]: 1) Direct immune cell suppression: Blebbisomes display high surface densities of PD-L1, which binds to PD-1 on effector T cells, inhibiting their activation. This suppressive effect may surpass that of exosomal PD-L1 due to the substantially larger cargo capacity of blebbisomes. 2) Blebbisomes often co-express multiple inhibitory molecules, such as PD-L1 and CD47, enabling them to simultaneously suppress T cell activity and evade macrophage-mediated clearance. Additionally, CD73 on the blebbisome surface enzymatically converts AMP into adenosine, which acts via A2A receptors to broadly inhibit immune cell function. Together, these features create a highly immunosuppressive tumor microenvironment. 3) Systemic impact: By trafficking to distant tissues, including lymph nodes and bone marrow, and releasing secondary EVs, blebbisomes may disrupt systemic antitumor immunity, extending their influence beyond the primary tumor site.

### Functional comparison with other lEV subtypes

Compared to LOs, blebbisomes contain functionally intact mitochondria, as confirmed by membrane potential staining, whereas LOs typically carry damaged or non-functional mitochondrial fragments [10]. Their biogenetic origins also differ: LOs arise from plasma membrane blebbing and often transport oncogenic cargo, while blebbisomes are derived from residual bodies formed during cell contraction and retain functional organelles. Consequently, blebbisomes resemble “miniaturized cells,” surpassing LOs in both metabolic capacity and sustained intercellular communication. In contrast to migrasomes and exophers, which primarily function in the clearance of cellular debris (e.g., damaged mitochondria and aggregated proteins), blebbisomes are specialized for active signal exchange [10]. Additionally, blebbisomes display independent motility and prolonged viability (72–96 h), enabling more extensive spatial and temporal influence [10].

In summary, blebbisomes represent the most structurally complex and functionally integrated EV subtype identified to date, reshaping our understanding of EV-mediated intercellular signaling. Their unique combination of structural complexity, diverse molecular cargo, and potent immunoregulatory capacity may unlock new opportunities in cancer immunotherapy, liquid biopsy, and targeted drug delivery. Future research priorities include mapping the cellular origins of blebbisomes, elucidating their dynamic interactions with immune cells, and validating their potential as therapeutic targets.

## Clinical potential of lEVs

As understanding of lEVs—particularly blebbisomes—continues to evolve, their clinical applications in tumor diagnosis and treatment are becoming increasingly prominent, especially in the following areas:

### Liquid biopsy biomarkers

Traditional tissue biopsies face inherent limitations, including sampling difficulty and tumor heterogeneity [22]. Large EVs, which are stably present in bodily fluids and enriched with tumor-specific molecules (e.g., mutant KRAS, methylated DNA, miRNAs), have emerged as ideal carriers for non-invasive biomarkers [31, 43, 154, 156]. Compared to small vesicles, lEVs offer larger size and more complex cargo profiles, making them particularly suitable for early cancer detection, tumor subtyping, and recurrence monitoring [157]. MBRs also hold promise as diagnostic biomarkers, as their quantification in liquid biopsies from cancer patients—relative to levels in normally cycling cells—may provide valuable clinical insights [73].

Numerous studies have highlighted the potential of lEVs for the early detection of thyroid cancer, ovarian cancer, and HCC [179–182]. For example, a mesoporous gold sensor-based electrochemical platform for non-small cell lung cancer (NSCLC) enables high-sensitivity detection of phosphorylated PD-L1 (pPD-L1) on EVs, providing a rapid, low-volume approach for immunotherapy assessment [31]. Similarly, the Exodisc system has efficiently separated large (EV-L) and small (EV-S) prostate cancer–derived EVs, with proteomic analysis revealing EV-L enrichment of PSMA-related markers, underscoring their diagnostic value [182].

Ahmed et al. performed a systematic analysis of plasma lEVs in patients with thyroid nodules, identifying significantly upregulated miR-195-3p and KLK11 in malignant cases compared with benign and healthy controls, highlighting their potential as biomarkers [179]. Importantly, the composition of lEVs varies widely among healthy individuals, with factors such as sex and age only partially accounting for this variability. Tetraspanin-positive lEVs predominantly originate from platelets, indicating complex release mechanisms and underscoring the need for standardized evaluation systems [183]. Furthermore, researchers emphasize the value of multi-marker panels: while single markers often lack sufficient sensitivity, combinations—such as EMMPRIN⁺/EpCAM⁺/MUC1⁺/EGFR⁺—can enhance the diagnostic AUC to 0.85 in breast cancer [18].

### Tumor-specific lEV subtype identification for precision early screening

The complexity and heterogeneity of lEVs continue to present challenges for their clinical application. Although a variety of EV isolation techniques—such as differential centrifugation, density gradient centrifugation, ultrafiltration, immunoaffinity capture, and microfluidic separation—have been developed, none achieve complete purification. Recently, several novel isolation and detection strategies have emerged. In pancreatic ductal adenocarcinoma (PDAC), researchers have identified specific lEV surface markers (e.g., CLDN4, TSPAN1, MUC5AC) to establish a highly sensitive detection platform that outperforms conventional markers such as CEA and CA19-9, while also allowing for postoperative monitoring [184]. In tumor-derived EVs (T-EVs), PD-L1 detection is often obscured by peripheral, non-tumor EVs. To address this, recent studies have employed dual-aptamer proximity ligation assays (PLA) combined with rolling circle amplification (RCA) to selectively detect EpCAM⁺PD-L1⁺ T-EVs, achieving a detection limit as low as 7.5 particles/μL in unpurified blood samples. This strategy demonstrates strong translational potential for early cancer screening and immunotherapy evaluation [185]. Building on this approach, the combination of multiple surface markers can significantly enhance the identification accuracy of specific lEV subtypes, enabling their high-precision isolation and broader clinical utility. A novel experimental protocol has also been developed that uses either 1.5% PEG6000 or PEG5000-coated gold nanoparticles to isolate MBRs or lEVs from mammalian cell culture medium. This method addresses the limitations of traditional ultracentrifugation, which is often costly and time-consuming [186].

### Therapeutic evaluation and recurrence monitoring

Dynamic changes in lEV quantity and composition reflect tumor responses to treatment. A significant reduction in tumor-derived lEV burden typically indicates effective therapy, whereas persistent or increasing levels suggest disease progression or the emergence of drug resistance [187, 188]. Tersigni et al. employed nano-flow cytometry to detect EpCAM⁺ and CD45⁺ EVs in venous blood, finding higher baseline levels in platinum-resistant patients and a marked increase 21 days post-treatment in chemotherapy-sensitive patients—demonstrating that lEVs can serve as dynamic indicators of chemotherapeutic response [180].

In solid tumor management, conventional imaging often fails to detect minimal residual disease (MRD). In HCC, the HCC EV TR Score system was developed, using click chemistry to enrich HCC-derived EVs and PCR to quantify six HCC-specific genes. This approach enabled real-time evaluation of treatment response and predicted recurrence up to 63 days earlier than MRI, achieving 88.2% sensitivity and 76.5% specificity [181]. Similarly, in high-grade serous carcinoma (HGSC), nanoparticle tracking analysis (NTA) of preoperative ascites and plasma EVs demonstrated strong correlations between lEV ratios and residual tumor burden following primary debulking surgery. Parameters such as mean diameter, D90 values, and medium/lEV ratios were consistent between ascites and plasma and effectively predicted postoperative residual disease and neoadjuvant chemotherapy (NACT) response [189]. These findings highlight the growing clinical value of lEVs in therapeutic evaluation and MRD monitoring, with size-dependent molecular profiling further elucidating their functional roles. Beyond oncology, lEVs are increasingly recognized in systemic disease monitoring. A large-scale, NIH-funded high-throughput proteomic study in healthy elderly individuals identified lEV subpopulations and proteomic signatures associated with human longevity. Immune cell-derived (HLA-ABC⁺, CD9⁺, CD31⁺) and skeletal muscle-derived (MCAD⁺, RyR2⁺) lEVs were enriched in long-lived individuals and carried complement proteins (e.g., C2, C6). A panel of 35 EV peptides accurately distinguished long-lived individuals from controls (AUC 0.91–1.00), suggesting their potential as predictive biomarkers for lifespan and aging [190].

Notably, the complement signaling axis may regulate immune homeostasis and aging via lEVs, providing a theoretical foundation for future anti-aging interventions. This study extends the clinical relevance of lEVs beyond oncology, emphasizing their role as “signal amplifiers” that integrate immunity, metabolism, and aging networks.

### Drug delivery vectors

lEVs exhibit excellent biocompatibility, membrane fusion capability, and immune evasion properties, making them highly attractive candidates for drug delivery systems [191]. Compared to cell-based therapies, lEV-based acellular approaches offer improved safety, greater controllability, and easier standardization. Blebbisomes, owing to their large size and structural complexity, can co-deliver multiple therapeutic agents—including small molecules, siRNA, and proteins—and can be engineered with tumor-targeting ligands via surface membrane modification [10, 191, 192]. Recent studies have highlighted several advantages of engineered lEVs over sEVs. Wang et al. (2025) reported that tumor-derived engineered lEVs exhibit higher protein and mRNA loading capacities, enhanced in vivo stability, improved organ targeting (e.g., liver and lung), and sustained therapeutic release. These properties make them ideal vehicles for mRNA vaccines and organ-targeted cancer immunotherapy [193]. ApoBDs also serve as promising delivery platforms by leveraging their intrinsic biological properties. One effective strategy involves pre-loading parental cells with therapeutic agents—such as antisense oligonucleotides (ASOs) or immunoadjuvant-modified nanorods (AuNR-CpG)—followed by induced apoptosis to generate cargo-loaded ApoBDs. Retaining the surface signatures of their cells of origin, ApoBDs derived from immune or tumor cells naturally achieve targeted delivery to homologous inflammatory sites or tumors [111, 171]. Yin et al. developed a novel nanoplatform for tumor therapy using MMP-2-responsive and PS-modified nanoparticles to deliver dasatinib, achieving precise targeting and efficient depletion of tumor-associated macrophages [194]. These engineered vesicles also demonstrate the innate ability to cross physiological barriers, including the blood–brain barrier (BBB), as shown by tumor cell-derived ApoBDs leveraging a CD44V6-mediated endothelial interaction mechanism to deliver ASOs and alleviate Parkinson’s disease in vivo. Additionally, they can target circulating monocytes or exploit tumor-homing macrophages for precise drug accumulation in solid tumors [195, 196]. Additionally, ApoBDs facilitate the “neighbor effect” by transferring therapeutics from peripheral apoptotic tumor cells to interior tumor regions, significantly enhancing drug penetration and overall tumor destruction [197]. Small ApoBDs (100–1000 nm) show particular promise due to the absence of DNA fragments and reduced phagocytic clearance, providing an efficient and adaptable approach for macromolecular drug delivery in oncology [195]. Similar benefits have been demonstrated in the treatment of neurological diseases. Patel et al. (2025) highlighted the potential of engineering cells to package therapeutic molecules into MBRs during mitosis by targeting MKLP1/KIF23. This strategy offers a promising avenue for developing novel drug delivery systems for a variety of diseases, including neurodegeneration and cancer [73]. Chen et al. (2025) demonstrated that engineered lEVs delivering functional mitochondria effectively restored energy metabolism in ischemic brain tissue. In these models, lEV-mediated mitochondrial transfer significantly boosted ATP production and reduced oxidative stress in oxygen-deprived brain endothelial cells, leading to improved cell survival—therapeutic effects not achievable with smaller sEVs. This approach could also be applied to cancer therapy by targeting drug resistance mechanisms through the delivery of metabolic pathway inhibitors or healthy mitochondria [192].

Moreover, beyond serving as delivery vehicles, certain lEV populations can intrinsically modulate tumor cell behavior. For instance, studies have shown that lEVs derived from NTERA2 teratocarcinoma cells can inhibit glioblastoma (GBM) cell migration by delivering the CRIPTO protein, without significantly affecting cell proliferation or chemotherapy sensitivity. This suggests that lEVs may also play a role in anticancer therapy by modulating tumor cell migration [12].

The intact membrane structures of lEVs provide enhanced fusion and signaling capabilities. For example, paclitaxel-loaded lEVs derived from lung cancer cells induce apoptosis via caspase-3 activation, inhibit autophagy through the Beclin-1/LC3 II/I pathway, and promote mitophagy via the Pink-1/Parkin pathway, thereby significantly improving therapeutic efficacy [198]. Plant-derived nanovesicles (PDNVs)—a non-mammalian form of lEVs—also exhibit promising antitumor effects. These vesicles, generally larger in size (200–1000 + nm), demonstrate excellent stability, biodegradability, and oral accessibility, making them particularly suitable for gastrointestinal cancer therapy. For instance, Platycodon grandiflorum-derived EVs (PGEVs) inhibit breast cancer growth by enhancing CD8⁺ T cell infiltration and reshaping the gut microbiome, providing a low-cost and high-safety option for clinical translation [199, 200]. Despite these advantages, lEVs face challenges regarding dosage control and safety. High-dose administration of MSC-derived lEVs can trigger tissue factor-dependent coagulation or pulmonary thrombosis following intravenous injection, highlighting the need for rigorous safety evaluation [201]. To improve drug solubility and bioavailability, researchers have combined lEVs with nanotechnology. For example, embedding curcumin-loaded lEVs into electrospun polyvinyl alcohol nanofibers has been shown to enhance drug encapsulation and targeting efficiency [202]. Additionally, stimulating cytokine production or overexpressing CD9 can increase both the yield and functionality of Wharton’s jelly MSC-derived lEVs, providing strategies for engineering optimization [203].

### Immunotherapy targets

Immunosuppressive lEVs released by tumors have emerged as promising therapeutic targets. Circulating levels of PD-L1⁺ EVs are negatively correlated with the efficacy of PD-1 antibody therapy [150]. Consequently, combining immunotherapy with strategies that target these EVs—such as nanocapture-mediated removal of PD-L1⁺ lEVs or disruption of EV–immune cell interactions—could substantially enhance treatment outcomes [167]. Another potential approach is to inhibit lEV biogenesis by targeting key molecules involved in vesicle formation. For example, suppressing NMIIB-mediated blebbisome production can reduce EV-induced immunosuppression, serving as a novel adjuvant strategy in tumor immunotherapy [167].

A recent molecular design strategy introduced an aggregation-induced emission (AIE) photosensitizer, TPETTBI, which targets mitochondria and combines photodynamic therapy (PDT) with photothermal therapy (PTT) to induce immunogenic cell death (ICD) and promote durable antitumor immunity [204]. Furthermore, migrasomes derived from human umbilical cord mesenchymal stem cells (MSCs), when internalized by dendritic cells, inhibit Th2 activation and cytokine production (IL-4, IL-5, IL-13) by suppressing RAGE signaling. In asthma models, this approach alleviated airway inflammation with greater stability and lower immunogenicity compared to direct MSC infusion [205]. Consistently, elevated levels of PD-L1⁺ lEVs are associated with poor responses to immunotherapy, further highlighting their role in immune resistance mechanisms [14]. The study also revealed a calcium-dependent mechanism by which monocytes form migrasomes that act as sustained-release reservoirs for cytokines such as TNF, IL-6, and IL-1β. This process is regulated by the tetraspanin Tspan9, enabling continuous cytokine secretion into the circulation. These findings position migrasomes as active mediators of localized inflammatory signaling, redefining their role from mere migration by-products to key modulators in monocyte-driven immune regulation [206].

### Unique potential of blebbisomes

Although still in the early stages of investigation, blebbisomes exhibit unique characteristics that open unprecedented clinical opportunities [9, 10]. Their high enrichment of immune checkpoint molecules may contribute to resistance against PD-1/PD-L1 monotherapy, highlighting the potential of combinatorial therapeutic strategies. For example, therapies that neutralize multiple inhibitory ligands carried by blebbisomes or inhibit their biogenesis through NMIIB suppression could significantly enhance the efficacy of immunotherapies [10]. Blebbisomes also hold promise as diagnostic tools. Their large size—approximately 1,000 times that of exosomes—enables them to carry abundant tumor-specific cargo, including proteins, DNA, and RNA, offering high specificity for liquid biopsy applications [16]. Detecting ultra-large EVs carrying combinations of tumor markers in blood samples may facilitate the precise identification of tumor-derived blebbisomes [10].

As drug delivery vehicles, blebbisomes offer several advantages. Their membrane composition, similar to that of target cells, enhances fusion efficiency, and their origin from diverse cell types—including cancer cells and fibroblasts—enables purification from sources such as mouse bone marrow [10]. These features support their translational potential in liquid biopsy, drug delivery, and immunomodulation. Furthermore, engineering blebbisomes with tumor-targeting ligands could improve both their precision and therapeutic efficacy. Their large cargo capacity makes them particularly well-suited for the co-delivery of multiple therapeutic agents.

## Isolation methods of lEVs

While sEVs have benefited from extensive validation, lEVs offer a potentially richer source of biomarkers and therapeutic agents due to their diverse cargo and functional complexity. However, overcoming challenges in isolation and standardization is critical to fully realize their potential. Efficient separation of lEVs remains a major barrier to both mechanistic studies and clinical translation. Their broad size heterogeneity, overlap with debris and lipoproteins, and the absence of subtype-specific markers mean that existing isolation techniques often yield mixed populations or fragmented lEVs, resulting in poor reproducibility. Consequently, the development of standardized, high-purity workflows is essential to comply with MISEV guidelines.

### Challenges in separating small and lEVs

EVs from different cellular origins—including exosomes, MVs, and oncosomes—often share overlapping size ranges and surface markers. sEVs (e.g., exosomes) generally exhibit narrower size distributions, making them easier to enrich using size-based strategies. In contrast, lEVs are larger and easier to detect, isolate, and standardize. Their size facilitates analysis via flow cytometry and microscopic imaging, whereas sEVs are often too small to be reliably detected on conventional clinical platforms, posing challenges for assay reproducibility and standardization. Nevertheless, serum-derived proteins and lipoproteins remain persistent sources of contamination, particularly during size-exclusion chromatography, where insufficient resolution can result in co-elution and functionally misleading outcomes. These challenges are further amplified by the diverse physicochemical properties of lEVs from different parental cells, which can lead to inconsistent recovery across isolation methods.

### Common detection and isolation methods

#### Ultracentrifugation-based approaches (UC, dUC, DGUC)

*Ultracentrifugation* (UC) remains a widely used method for recovering EVs from biofluids such as plasma, breast milk, cerebrospinal fluid (CSF), urine, and cell culture supernatants [207, 208].

*Differential ultracentrifugation (dUC)* employs sequential centrifugation speeds (300–500 × g → 10,000–20,000 × g → 100,000–120,000 × g) to enrich EVs based on their sedimentation rates [209]. Advantages of dUC include operational simplicity, scalability, and relatively high yield. However, high shear forces may damage vesicles, cause aggregation, or result in loss, and large vesicles can become trapped within debris during low-speed steps.

*Density-gradient ultracentrifugation (DGUC)* offers significantly higher purity and improved subtype separation by exploiting buoyant density. The method is, however, time-consuming and typically yields fewer particles [208]. A combined approach—rapid enrichment by dUC followed by DGUC refinement—is often adopted to balance throughput with sample integrity [210].

#### Size-based techniques (SEC, TFAC, ultrafiltration)

*Size-exclusion chromatography (SEC)* separates particles based on differential pore entry, enabling gentle, non-destructive isolation while preserving vesicle bioactivity [211, 212]. Although SEC provides high resolution, its effectiveness decreases for EV subtypes of similar size and often results in dilution or incomplete removal of serum proteins.

*Tangential Flow for Analyte Capture (TFAC)* mploys ultrathin nanoporous membranes under tangential flow to selectively retain EVs through a capture–wash–release sequence [213]. Compared with conventional filtration, TFAC minimizes fouling, preserves vesicle morphology under low pressure, and exhibits strong “pore-edge capture” efficiency for lEVs. Nevertheless, heterogeneous lEV populations and pore clogging remain challenges, often necessitating additional purification steps.

*Ultrafiltration (UF)* uses membranes with defined pore sizes or molecular weight cut-offs (MWCOs) to rapidly concentrate EVs [214–216]. While cost-effective and gentle, UF can suffer from membrane fouling, co-retention of debris and protein aggregates, and variability arising from differences in membrane material or applied pressure. For lEVs, the formation of a “cake layer” can further reduce purity, highlighting the need for complementary downstream purification methods [215, 217].

#### Precipitation-based methods

*Polyethylene glycol (PEG)* reduces EV solubility to induce precipitation, enabling low-cost, equipment-independent enrichment from large sample volumes [218]. While this method achieves high recovery—including large vesicles—it is prone to co-precipitation of proteins, lipoproteins, and polymer residues. Consequently, subsequent purification using SEC or density-gradient centrifugation is typically required for reliable downstream analyses [219].

#### Immunoaffinity capture

Immunoaffinity capture exploits antibody–antigen interactions to selectively enrich EV subsets expressing markers such as CD63, CD81, CD9, EpCAM, or platelet-specific markers CD61/CD41 [207, 220]. Due to their large surface area, lEVs presenting high-copy antigens exhibit strong multivalent binding, enabling selective enrichment and discrimination within heterogeneous lEV populations. However, the lack of consensus on lEV-specific markers introduces potential subtype bias. Additionally, the high cost and low throughput limit this method to secondary purification rather than bulk isolation.

### Comparative evaluation and method selection

No single isolation strategy simultaneously achieves high purity, high yield, and structural preservation for lEVs. As a result, combined workflows—such as dUC followed by DGUC, SEC coupled with immunoaffinity capture, or TFAC integrated with SEC—represent the most robust approaches for isolating intact, well-defined lEV populations suitable for mechanistic and translational studies. A summary of commonly used lEV isolation techniques and their main characteristics is provided in Table 3.

**Table 3:** Comparison of lEV isolation methods

Method	Principle	Advantages	Disadvantages
UC	Sedimentation under high centrifugal force; separation by size and density	Widely used; suitable for various biofluids; scalable for large volumes	High shear may damage vesicles; aggregation or loss; co-sedimentation with debris
dUC	Sequential centrifugation at increasing g-forces (300–500 × g → 10,000–20,000 × g → 100,000–120,000 × g), separating by sedimentation rate	Simple; scalable; relatively high yield	Vesicle deformation; lEVs may become trapped in debris; limited purity
DGUC	Separation based on buoyant density in gradient media	High purity; improved subtype resolution	Time-consuming; lower particle yield
SEC	Separation by differential pore entry; larger EVs elute first	Gentle, non-destructive; preserves bioactivity; high resolution	Ineffective for similarly sized EVs; sample dilution; incomplete removal of serum proteins
TFAC	Ultrathin nanoporous membranes under tangential flow enabling capture–wash–release	Reduced fouling; high selectivity; low pressure preserves morphology; effective for lEVs	Pore clogging; heterogeneity requires additional purification
UF	Membranes with defined pore sizes or MWCOs retain larger particles	Fast; low cost; gentle; no ultracentrifuge required	Membrane fouling; co-retention of debris/protein aggregates; variability due to membrane material and pressure
PEG Precipitation	Volume exclusion reduces EV solubility and induces precipitation	Simple; low-cost; no specialized equipment; high recovery including lEVs	Low purity; co-precipitation of proteins/lipoproteins/polymers; requires secondary purification
Immunoaffinity Capture	Antibody–antigen recognition on beads, chips, or columns	High specificity; high purity; subtype enrichment based on markers	Marker heterogeneity; subtype bias; low throughput; high cost

Despite their tremendous potential, several challenges must be addressed before clinical translation can be realized: 1) Standardization: Efficient purification, molecular characterization, and large-scale production of engineered lEVs remain technically complex. 2) Specificity: Differentiating tumor-derived EVs from those released by healthy cells, while accounting for inter-patient heterogeneity, will require personalized strategies. 3) Safety: High-dose administration of MSC-derived lEVs may carry risks, including tissue factor–dependent coagulation or pulmonary thrombosis, highlighting the need for rigorous safety evaluation.

## Future perspectives

Despite the remarkable potential of lEV subtypes (e.g., blebbisomes) in tumor biology and clinical applications, several critical challenges remain in elucidating their fundamental mechanisms and advancing translational utility. Future research should prioritize the following key directions:

### Development of high-purity, high-throughput isolation techniques

Current mainstream isolation methods—such as ultracentrifugation and nanofiltration—have limited capacity to effectively separate large lEVs, particularly blebbisomes. Most commercial extraction kits are optimized for sEVs, hindering in-depth investigation of lEVs. However, recent advances in detection and characterization technologies are expanding their use in both basic and clinical research. Single-particle analysis techniques, including nano-flow cytometry, tunable resistive pulse sensing (TRPS), electron microscopy, and label-free optical imaging, have significantly improved resolution for characterizing lEVs heterogeneity and function [221]. For example, a study using Expi293F-engineered cells established a multi-scale analytical pipeline that revealed cargo distribution differences from population-level down to single-molecule resolution and identified high-efficiency cargo-loading structures. When combined with advanced imaging technologies such as dark-field microscopy, interferometric scattering microscopy (iSCAT), and surface plasmon resonance microscopy (SPRM), it is now possible to track lEVs and their receptor interactions in real time, providing a solid technical foundation for tumor diagnostics, drug delivery, and lEV engineering [222, 223].

A novel ruthenium complex fluorescent dye (Rubb7-TNL/Rubb7-TL) has been developed to specifically label lEVs, allowing clear distinction from cellular membrane debris. These dyes exhibit strong phosphorescent stability and consistent staining across multiple sources (e.g., A549, THP-1), likely due to RNA recognition within the vesicles. Interestingly, lipopolysaccharide-stimulated THP-1 cells release more and larger lEVs with unique biochemical profiles compared to resting cells [224]. Future research should integrate microfluidics and biorecognition chip technologies to develop multidimensional platforms that combine physical parameters and surface markers for high-throughput, precise enrichment of specific EV subtypes. Additionally, lEV composition is influenced by physiological states such as metabolism. A study in healthy individuals revealed postprandial changes in the lEV proteome, including upregulation of transport-related and epithelial/endothelial proteins, as well as increased CD324 (E-cadherin) levels detected via flow cytometry. These findings suggest that lEVs participate in rapid postprandial physiological responses, potentially originating from epithelial cells, and emphasize the importance of controlling fasting state in EV studies [225].

### Multi-omics functional analysis at single-vesicle resolution

Given the heterogeneity of lEVs, single-vesicle proteomics, transcriptomics, and metabolomics are essential for elucidating the molecular composition, biological functions, and tumor-promoting mechanisms of specific subtypes, such as LOs, migrasomes, and blebbisomes. This approach will also clarify their potential as therapeutic targets across different cancer types.

### Mechanisms of blebbisomes trans-tissue migration and immune interactions

The in vivo migration pathways of blebbisomes and their interactions with immune organs (e.g., lymph nodes and bone marrow) remain largely unknown. Advanced imaging techniques, such as correlative light and electron microscopy (CLEM), combined with blebbisome-specific tracking systems, are urgently needed to visualize their tissue penetration, directional accumulation, and intercellular signaling dynamics in real time.

### In-depth lipidomics and metabolic network studies

Although significant progress has been made in lEV proteomics and nucleic acid profiling, lipidomics remains underexplored. Future research should focus on the roles of specific lipids in vesicle biogenesis, membrane stability, signal transduction, and energy reprogramming to fully understand the metabolic functions of lEVs.

### Engineering and clinical translation pathways

Realizing the clinical potential of lEVs requires scalable engineering and production methods. Natural EVs face three primary obstacles: low yield, limited bioactivity, and poor targeting. Tumor-derived ApoBDs pose significant challenges for therapeutic applications due to their inherent procoagulant activity, primarily mediated by surface-exposed tissue factors and PS [226]. This property not only elevates the risk of venous thromboembolism in cancer patients [227] but also contributes to their immunogenicity. Consequently, comprehensive safety assessments—including coagulation monitoring—are essential during preclinical and clinical development. While surface engineering may help mitigate these procoagulant effects, such intrinsic biological activities highlight important limitations for their use as drug delivery carriers. Several strategies have been proposed to address these issues. For instance, membrane extrusion can improve yield, TS-induced miR-21 upregulation can enhance functional activity, and ALN surface modification can promote bone targeting [228]. To optimize lEV production, a 3D-printed perfusion bioreactor has been developed, increasing macrophage-derived exosome yield 12.5-fold while enhancing osteogenic and angiogenic capabilities. Mechanistically, fluid shear stress activates Piezo1 channels, triggering Ca^2^⁺ influx and YAP nuclear translocation, thereby promoting M2 polarization and EV release. Incorporating these bioreactor-derived EVs into electrospun membrane–hydrogel composites demonstrated superior regenerative capacity in a rat calvarial defect model, providing a cost-effective and scalable platform for clinical translation [228].

In the context of complex tumor-targeting requirements, lEV surface engineering is particularly important. For example, expressing LAMP2b fused with RGD or iRGD peptides enhances tumor targeting, while chemical modifications and pH-responsive coatings further improve delivery stability and specificity within the tumor microenvironment. Compared to sEVs, lEVs possess a richer repertoire of membrane proteins and larger spatial structures, making them ideal carriers for immunomodulatory agents or RNAi therapeutics. The systematic development of multimodal, controlled-release, and actively targeted lEV platforms may revolutionize precision cancer therapy [192]. Large EVs also hold promise in intraoperative navigation. The ONCOGREEN strategy, for instance, utilizes autologous plasma-derived large EVs (PDEVs) loaded with the near-infrared dye indocyanine green (ICG) for real-time tumor margin imaging. In mouse models, this approach achieved excellent targeting precision, deep tissue penetration, and high-resolution imaging without toxicity. Furthermore, a large-scale PDEV extraction and lyophilization protocol based on plasmapheresis has been developed, laying the groundwork for clinical applications in surgical navigation and drug delivery [176].

## Conclusion

Large EVs expand our understanding of EV-mediated communication by encompassing a diverse spectrum of biogenesis pathways, cargo compositions, and context-specific functions that complement those of sEVs. While sEVs remain the most extensively studied and standardized EV subtype, lEVs possess several distinctive advantages. Their substantially larger size allows the transfer of structurally complex payloads—including intact mitochondria, multi-protein assemblies, cytoplasmic fragments, and high-molecular-weight RNAs—that sEVs cannot readily accommodate. This unique cargo capacity underpins many of the biological functions discussed in this review, such as enhanced metabolic rescue, organelle quality control, tumor microenvironment remodeling, and immunomodulation. Furthermore, multiple lEV subtypes, including blebbisomes, migrasomes, exophers, and MBRs, exhibit specialized roles that extend beyond classical exosome-mediated signaling.

Compared with sEVs, lEVs also offer several practical advantages for mechanistic research and translational applications. Their larger size facilitates detection and phenotyping via flow cytometry, fluorescence microscopy, and emerging high-resolution imaging platforms, whereas sEVs often fall below the sensitivity thresholds of conventional assays. Large EVs also tend to display richer and more diverse surface protein repertoires, enhancing subtype discrimination and enabling more reliable immunophenotyping. Additionally, the preservation of intracellular structures within certain lEV subtypes provides a more integrated reflection of parental-cell metabolic states, stress responses, and organelle integrity—features not typically captured by sEVs.

Despite these strengths, significant challenges remain. Large EV heterogeneity, overlap with apoptotic debris, and the absence of subtype-specific markers complicate isolation and mechanistic interpretation. Achieving high-purity lEV preparations requires refined separation workflows and improved standardization, particularly in comparison with the more established methodologies available for sEVs. Comprehensive multi-omics profiling, deeper characterization of biogenesis pathways, and advances in single-vesicle analytics will be essential for determining when lEVs provide redundant, complementary, or uniquely valuable insights relative to sEVs. Collectively, these efforts are crucial for unlocking the full diagnostic and therapeutic potential of lEVs and for establishing them as a distinct and biologically meaningful component of the EV field.

## Data Availability

No datasets were generated or analysed during the current study.

## Abbreviation

ABCC1: ATP Binding Cassette Subfamily C Member 1
AKT: AKT Serine/Threonine Kinase
Alix: ALG-2-Interacting Protein X
ApoBDs: Apoptotic Bodies
ApoEVs: Apoptotic Extracellular Vesicles
ApoMVs: Apoptotic Microvesicles
ApoExos: Apoptotic Exosomes
ARF6: ADP-Ribosylation Factor 6
ARMMs: Arrestin Domain–Containing Protein 1–Mediated Microvesicles
ATG5/ATG7: Autophagy Related Protein 5 / 7
ATP: Adenosine Triphosphate
ATP6V1G1: V-ATPase Subunit G1
BBB: Blood–Brain Barrier
BDNF: Brain-Derived Neurotrophic Factor
BSG/CD147: Basigin
CAFs: Cancer-Associated Fibroblasts
CCL2/CCL12: C-C Motif Chemokine Ligand 2 / 12
CD9: Tetraspanin CD9
CD47: CD47 Molecule
CD63: Tetraspanin CD63
CD81: Tetraspanin CD81
CD206: Mannose Receptor C-type 1
circRNA: Circular RNA
CLEM: Correlative Light and Electron Microscopy
COL12A1: Collagen Type XII Alpha 1 Chain
CTLs: Cytotoxic T Lymphocytes
CTLA-4: Cytotoxic T-Lymphocyte–Associated Protein 4
CXCL12: C-X-C Motif Chemokine Ligand 12
DCs: Dendritic Cells
DHCR7: 7-Dehydrocholesterol Reductase
DIAPH3: Diaphanous-Related Formin 3
DNA: Deoxyribonucleic Acid
Drp1: Dynamin-Related Protein 1
EFNB: Ephrin-B Ligand
ENDS: Elongated Neutrophil-Derived Structures
ENO1: Enolase 1
EphB: Ephrin Type-B Receptor
ERK: Extracellular Signal-Regulated Kinase
ESCRT: Endosomal Sorting Complex Required for Transport
EVs: Extracellular Vesicles
FASL: Fas Ligand
FDCs: Follicular Dendritic Cells
FGF: Fibroblast Growth Factor
GAPDH: Glyceraldehyde-3-Phosphate Dehydrogenase
GLS: Glutaminase
HCC: Hepatocellular Carcinoma
HGSC: High-Grade Serous Carcinoma
HLA-E: Human Leukocyte Antigen–E
HSPD1/HSP60: Heat Shock Protein Family D Member 1 / 60 kDa Heat Shock Protein
IL-6/IL-8/IL-33: Interleukin-6 / 8 / 33
ILVs: Intraluminal Vesicles
IR: Insulin-Resistant
KRAS: KRAS Proto-Oncogene
LAND-Vs: Large Aging Neutrophil-Derived Vesicles
LC3/LC3B: Microtubule-Associated Protein 1 Light Chain 3
LDHB: Lactate Dehydrogenase B
lEVs/Large EVs: Large Extracellular Vesicles
lncRNA: Long Non-Coding RNA
LOs: Large Oncosomes
MAPK: Mitogen-Activated Protein Kinase
MBs: Midbodies
MBRs: Midbody Remnants
MDVs: Mitochondrial-Derived Vesicles
MET: MET Proto-Oncogene, Tyrosine Kinase
miRNA: MicroRNA
miR-1227 etc.: Specific microRNAs (miR-1227, miR-155, miR-21, etc.)
MISEV: Minimal Information for Studies of Extracellular Vesicles
MitoEVs: Mitochondrial Extracellular Vesicles
Mito-lEVs: Mitochondria-Enriched Large Extracellular Vesicles
MKLP1/KIF23: Mitotic Kinesin-Like Protein 1
MLCK: Myosin Light Chain Kinase
MLC: Myosin Light Chain
MMP2/MMP9: Matrix Metalloproteinase-2 / − 9
MUC1: Mucin 1
MVBs: Multivesicular Bodies
MYC: MYC Proto-Oncogene
NACT: Neoadjuvant Chemotherapy
NAFs: Normal-Associated Fibroblasts
NK cells: Natural Killer Cells
NKG2: Natural Killer Group 2 Receptor
NMIIA/NMIIB: Non-Muscle Myosin IIA / IIB
NNMT: Nicotinamide N-Methyltransferase
Nrf2: Nuclear Factor Erythroid 2–Related Factor 2
NSCLC: Non–Small Cell Lung Cancer
NTA: Nanoparticle Tracking Analysis
PAM: Peptidylglycine Alpha-Amidating Monooxygenase
PANX1: Pannexin-1
Parkin: E3 Ubiquitin-Protein Ligase Parkin
PD-1: Programmed Death 1
PD-L1/PD-L2: Programmed Death-Ligand 1 / 2
PDAC: Pancreatic Ductal Adenocarcinoma
PI(4,5)P₂: Phosphatidylinositol-4,5-Bisphosphate
PINK1: PTEN-Induced Kinase 1
PKM1/PKM2: Pyruvate Kinase M1/M2
PLEXIN-B2: Plexin B2
PNI: Perineural Invasion
PP2A: Protein Phosphatase 2A
PSMB4: Proteasome Subunit Beta Type-4
PTP1B: Protein Tyrosine Phosphatase 1B
PS: Phosphatidylserine
PTEN: Phosphatase and Tensin Homolog
Rab GTPases: Ras-Related Rab Family GTPases
RAB22A: Ras-Related Protein Rab-22A
ROCK: Rho-Associated Coiled-Coil Containing Protein Kinase
RNF146: Ring Finger Protein 146
RUVBL2: RuvB-Like AAA ATPase 2
SDC4: Syndecan-4
SERPINA12: Serpin Family A Member 12
SREBP2: Sterol Regulatory Element-Binding Protein 2
SPRM: Surface Plasmon Resonance Microscopy
STAT3: Signal Transducer and Activator of Transcription 3
sEVs: Small Extracellular Vesicles
STEAP1/2: Six-Transmembrane Epithelial Antigen of the Prostate 1/2
T cells: T Lymphocytes
TR Score: Treatment Response Score
TFEB: Transcription Factor EB
TGF-β: Transforming Growth Factor Beta
TGM2: Transglutaminase 2
TMPs: T cell Microvilli Particles
TNF: Tumor Necrosis Factor
TRAIL: TNF-Related Apoptosis-Inducing Ligand
TUFM: Tu Translation Elongation Factor, Mitochondrial
uPAR: Urokinase Plasminogen Activator Receptor
V-ATPase: Vacuolar-Type H + -ATPase
VAMP3: Vesicle-Associated Membrane Protein 3
VAPA: VAMP-Associated Protein A
VE-cadherin: Vascular Endothelial Cadherin
VEGF: Vascular Endothelial Growth Factor
